# EST–SNP Study of *Olea europaea* L. Uncovers Functional Polymorphisms between Cultivated and Wild Olives

**DOI:** 10.3390/genes11080916

**Published:** 2020-08-10

**Authors:** Roberto Mariotti, Angjelina Belaj, Raul De La Rosa, Lorenzo Leòn, Federico Brizioli, Luciana Baldoni, Soraya Mousavi

**Affiliations:** 1 CNR—Institute of Biosciences and Bioresources, Via Madonna Alta 130, 06128 Perugia, Italy; roberto.mariotti@ibbr.cnr.it (R.M.); fbrizioli@gmail.com (F.B.); soraya.mousavi@ibbr.cnr.it (S.M.); 2 IFAPA—Centro Alameda del Obispo, Avda Menendez Pidal, s/n, E-14004 Cordoba, Spain; angjelina.belaj@juntadeandalucia.es (A.B.); raul.rosa@juntadeandalucia.es (R.D.L.R.); lorenzo.leon@juntadeandalucia.es (L.L.)

**Keywords:** *Olea europaea* subsp. *europaea*, subsp. *guanchica*, cultivars, oleasters, EST–SNPs, candidate genes, functional markers, genomics-assisted breeding, conservation

## Abstract

Background: The species *Olea europaea* includes cultivated varieties (subsp. *europaea* var. *europaea*), wild plants (subsp. *europaea* var. *sylvestris*), and five other subspecies spread over almost all continents. Single nucleotide polymorphisms in the expressed sequence tag able to underline intra-species differentiation are not yet identified, beyond a few plastidial markers. Methods: In the present work, more than 1000 transcript-specific SNP markers obtained by the genotyping of 260 individuals were studied. These genotypes included cultivated, oleasters, and samples of subspecies *guanchica*, and were analyzed in silico, in order to identify polymorphisms on key genes distinguishing different *Olea europaea* forms. Results: Phylogeny inference and principal coordinate analysis allowed to detect two distinct clusters, clearly separating wilds and *guanchica* samples from cultivated olives, meanwhile the structure analysis made possible to differentiate these three groups. Sequences carrying the polymorphisms that distinguished wild and cultivated olives were analyzed and annotated, allowing to identify 124 candidate genes that have a functional role in flower development, stress response, or involvement in important metabolic pathways. Signatures of selection that occurred during olive domestication, were detected and reported. Conclusion: This deep EST–SNP analysis provided important information on the genetic and genomic diversity of the olive complex, opening new opportunities to detect gene polymorphisms with potential functional and evolutionary roles, and to apply them in genomics-assisted breeding, highlighting the importance of olive germplasm conservation.

## 1. Introduction

Olive (*Olea europaea* L.) is a long-living widespread tree species. Its genetic patrimony includes thousands of cultivated varieties, wild olives, and related subspecies, such as *cuspidata, laperrinei*, *cerasiformis, maroccana,* and *guanchica,* which grow in natural areas of different continents [1,2,3,4,5]. The cultivated and wild (also named oleaster) forms of olive (subsp. *europaea*, var. *europaea* and *sylvestris*, respectively), typical of the Mediterranean area, are diploid, with 23 chromosomes and a genome size of about 1.48 Giga base pairs [6]. Subspecies *guanchica* is a diploid subspecies spreading only in the Canary Islands [7].

The high genetic variability of cultivated olive [8,9,10,11,12] (mainly related to its allogamy), the lack of turnover with modern cultivars, a remarkable tree longevity, and the capacity to survive out of managed cultivation, allowed it to spread over a wide range of environments [13,14,15,16]. Inside this diversified scenario, the debate on olive domestication and the contribution of oleasters and related subspecies to this process, is still open and controversial [17,18,19,20,21,22]. Some recent analyses suggest that the olive tree domestication occurred in the Levant (Syrian–Turkish) area, followed by secondary diversifications in two opposite directions, to the west side across the shores of the Mediterranean [13,23,24,25] and up to the east in the Iranian plateau [16,26,27]. Recently it was reported that the gene flow between domesticated olives and the wild relatives allowed secondary diversification and contributed to the emergence of thousands of local varieties [15,18].

During plant domestication, wild species were converted into crop plants through artificial selection, with conscious and unconscious contribution [28]. Artificial selection might influence important loci variation, affecting traits of agronomic interest. Artificially selected SNPs might be consistently linked to important agronomic traits [29]. Artificial selection of crop varieties focused on modifying factors such as plant architecture and stress resistance, as a means of increasing yield [30]. The selection process in cereal crops left observable genetic changes, including a reduction of genetic diversity and an increased frequency of favorable seed size alleles in the cultivated lines, as compared to their wild progenitors [31,32,33]. The artificial selection could also be the cause of rapid demographic expansions, as reported for the cotton cultivation [34].

Domestication of perennial plants is expected to differ from that of annual plants, as a consequence of the evidence that trees are usually propagated vegetatively and have an extended juvenile stage, thus domestication bottleneck cannot be as severe as that for annual plants [35]. Considering what was reported previously, perennial crops retain, 95% of neutral variation from their progenitors, on average, while the annuals only retain 60% [36]. There are only few works related to perennial fruit trees. In grape, differentiation between wild and cultivated plants caused morphological changes on berry size, sugar content, seed morphology, and a shift from dioecy to hermaphroditic mating system [35,37]. Recently, a transcriptomic analysis on wild and cultivated olives indicated that the domestication of olives had moderate genomic consequences and was more effective on gene expression [38]. The studies on selection signatures might provide a “bottom-up” approach [39] on the genetic basis that differentiate the present cultivated plants from wild trees [22]. In fact, cultivated olives were introduced from the eastern side of the Mediterranean basin, as seedlings, grafting, and cuttings, strongly reducing the genetic diversity carried by autochthonous olives, such as the central-western oleasters [23].

Sequences related to expressed tags such as the long core repeat of EST–SSRs [40,41,42], developed from transcript collections, might contribute to start the study of gene differences between olive subspecies, highlighting the possible functional effect of polymorphisms between olive genotypes [42]. In the last years, special attention was paid to the development and use of SNP markers in olive [43,44,45,46,47] and, thanks to a wide set of EST–SNPs recently developed [48], the study of functional polymorphisms in olive is finally having a boost. The gene flow between oleasters, subspecies *guanchica* and Western Mediterranean and some cultivated varieties was already reported by using a different set of markers [3,49,50]. Therefore, based on the evidence of the previous studies, this work aimed to identify potential functional markers for differentiating cultivated from wild olive trees, by addressing the following questions—(a) what genes distinguish cultivars from oleasters and *guanchica* olives? (b) what are the functions of differently evolved genes? (c) which of these gene polymorphisms could be introgressed into cultivated varieties through genomic-assisted breeding, to improve their performance?

## 2. Materials and Methods

### 2.1. Plant Material and EST—SNP Genotyping

Data of 156 olive cultivars, 73 wild olives and 16 *guanchica* samples from the World Olive Germplasm Collection of IFAPA (Córdoba, Spain), were collected from a previous published study [47], while 15 cultivars from the International Olive Germplasm Collection of Lugnano in Teverina (Terni, Italy), were de-novo genotyped using the same approach [47]. The final selection of common markers included 1040 EST—SNPs for 260 genotypes (Table S1).

### 2.2. Genetic Differentiation and Population Genetic Structure

General relationships within populations and amongst populations were described through a principal coordinate analysis (PCoA) performed by GenAlEx 6.5, based on a covariance matrix created from pairwise genetic distances [51], while the three-dimensional visualization was made by the online tool Cubemaker (https://tools.altiusinstitute.org/cubemaker/). Phylogenetic analyses were performed using a parametric method based on sequence alignment data. All SNP allele’s calls for the 260 genotypes were concatenated to give a specific sequence for each accession, using the IUPAC code. Multiple sequence alignment was performed using ClustalW in the Bioedit software [52], and the files were converted into MEGA format using MEGAX [53]. A phylogenetic tree was constructed with MEGAX, with a bootstrap inferred from 1000 replicates. The optimal tree had a sum of branch length equal to 6.34. The tree was drawn to scale, with branch lengths in the same units as those of the evolutionary distances used to infer the phylogenetic tree. The evolutionary distances were computed using the Maximum Composite Likelihood method and were in the units of the number of base substitutions per site. All positions with less than 95% site coverage were eliminated. Alignment gaps lower than 5%, missing data, and ambiguous bases were allowed at any position. This analysis involved 260 nucleotide sequences and 1040 positions in the final dataset. The phylogenetic tree was drawn and visualized using FigTree v1.4.3 (http://tree.bio.ed.ac.uk/software/figtree/).

The genotypic data were analyzed using the Bayesian clustering algorithm of STRUCTURE v2.3 [54,55], using the admixture model and correlated allele frequencies with a burn-in of 100,000 iterations, run length of 100,000, and K = 2 to 10. The optimal value of K was determined using the delta K procedure, using STRUCTURE Harvester [56].

### 2.3. Genetic Diversity

The genetic diversity was calculated by using DnaSP 6 [57]. Polymorphic sites and DNA polymorphisms were detected for the overall sample set, while DNA diversity, polymorphism and divergence, gene flow, genetic differentiation, and population size changes were done within and between the three selected populations. Nucleotide diversity based on the average pairwise difference among sequences (Pi) and the average number of nucleotide differences (k) were also calculated by DnaSP 6. In order to detect if the three selected populations evolved randomly or the mutations were under selection pressure, Tajima’s D test was also performed. The obtained diversity indices were strictly related to the EST–SNPs described previously.

Heterozygosity, F statistics, Polymorphism by Population, Allele Frequencies by Population (AFP), and Allele Frequencies by Population for the Haploid Data (HAFP), were performed by GenAlEx 6.5.

### 2.4. Identification and Functional Analysis of EST–SNP

The set of SNPs, derived from the expressed sequence tags, and each nucleotide change could correspond to a different level of expression or protein structural variation, both for synonymous (sSNP) and non-synonymous (nsSNP) SNPs. All EST–SNPs were compared between the three olive groups and a cut-off of 45% of frequencies between two homozygous states was selected (e.g., SNP1 frequency: AA_cultivated_ minus AA_wild_ ≥45% yes, <45% no). All transcripts showing two different homozygosity states for the cultivated group *versus* oleasters and subsp. *guanchica*, were classified. The sequences of each contig were blasted into the NCBI database, using the BLASTN algorithm of the NCBI-blast procedure (https://blast.ncbi.nlm.nih.gov/), in order to identify nucleotide polymorphisms on the gene sequences. For the uncharacterized genes, the predicted amino acid sequences were used as a query in the same website, using BLASTP. In order to annotate the contigs that were differently allocated among the three groups of genotypes, they were used as query to blast in the NCBI database. Nucleotide changes were investigated using the ExPASy translate tool (http://web.expasy.org/translate/) for both alleles, in order to identify potential amino acid variations. Finally, for the functional classification according to Gene Ontology (GO) terms, the identified genes were used as a query for a statistical overrepresentation test, using the PANTHER online tool [58], which gave the *Arabidopsis thaliana* reference list with FISHER test type (http://pantherdb.org/tools/compareToRefList.jsp). In order to categorize the biological processes, the cellular component and the molecular function of each gene, the search was also performed on TAIR (https://www.arabidopsis.org/) and UniProtKB (https://www.uniprot.org/uniprot) web tools, including all plant species.

## 3. Results

### 3.1. Genetic Differentiation and Population Structure

**Figure 1:**
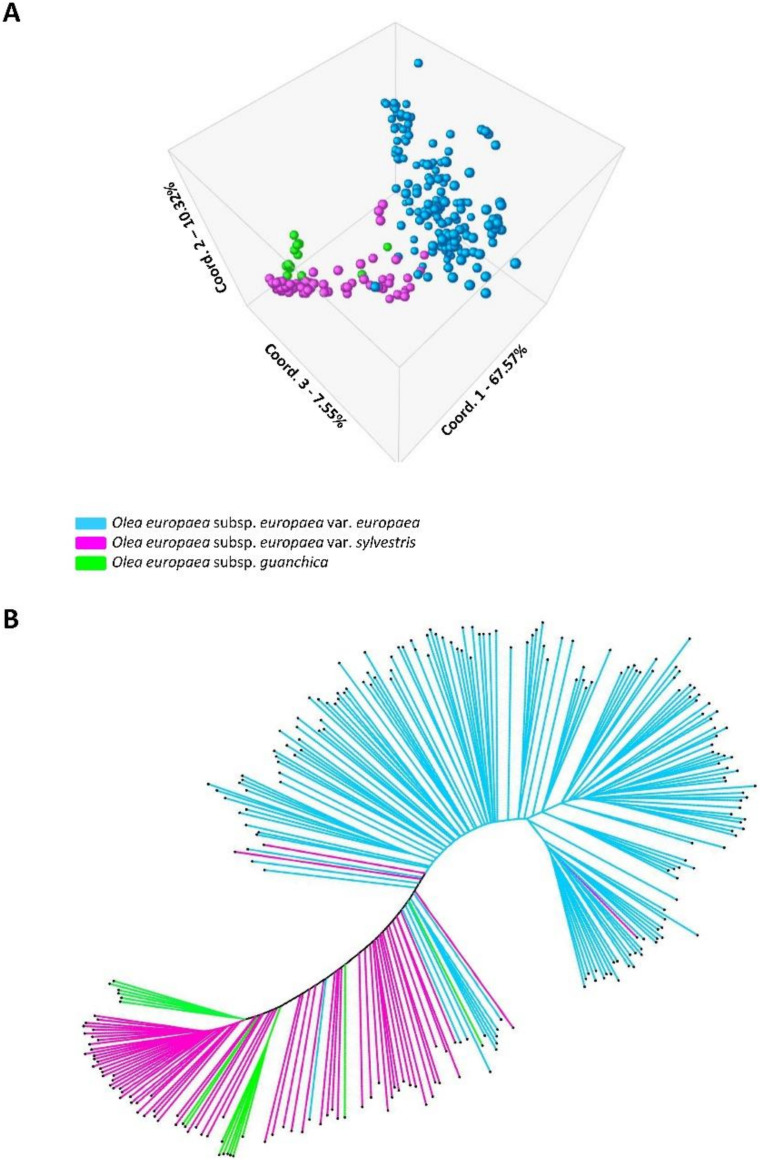
The Principal Coordinate (PCoA), 2- and 3-dimensional graphs, analysis based on a genetic distance matrix between the 260 olive genotypes (**A**); unrooted neighbor joining tree constructed with MEGA X of 260 olive genotypes (**B**). Colors representation, light blue—cultivated varieties; purple—oleaster; green—subsp. *guanchica.*

Based on the results of the genetic differentiation and population structure analyses, the genetic diversity analyses were performed by dividing the sample set in three populations (cultivars, oleasters, and *guanchica* samples). The PCoA analysis (Figure 1A), based on a genetic distance matrix between the 260 olive genotypes, the three coordinates (1, 2, and 3) corresponding to the x, y, and z axes, represented the 67.57% and the 10.32% and 7.55% of total variability, respectively. It showed a clear separation between the cultivars and the group of oleaster and subsp. *guanchica*, with only a few mixed samples between the wild and the cultivated groups. In the first case, a supposed oleaster more likely represented a feral form (seedling from a cultivated olive), meanwhile in the latter case, the cultivated olive showed a strong wild genetic background. In fact, an interesting overlap in the crossing of axes among the cultivars and the oleasters was observed, evidencing a clear introgression of gene polymorphisms from var. *sylvestris* into var. *europaea*. The resultant *guanchica* samples were intermixed with the oleasters and quite distant from the cultivated genotypes.

The Unrooted Neighbor Joining tree clearly confirmed the differentiation between cultivars and oleasters (Figure 1B). This elaboration showed some *guanchica* plants together with oleaster, even if, almost two separated sub-clusters could be observed. Seven olive cultivars clustered within the var. *sylvestris*’s group, while only one oleaster, putatively a cultivar seedling, was placed within the cultivar’s group after both analyses.

**Figure 2:**
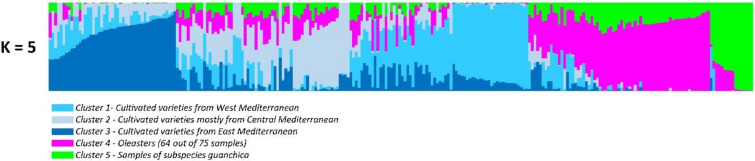
Estimate of genetic diversity of 260 *Olea europaea* accessions analyzed by 1040 SNP markers. Bar-plot describes the population structure estimated by the Bayesian clustering. According to the most informative K values, five clusters were distinguished. Each individual was represented by a horizontal colored line, with a length proportional to the estimated membership coefficient. Clusters 1, 2, and 3 represent the three sub-populations of the cultivated varieties: Cluster 1 (light blue)—almost all cultivated varieties from West Mediterranean, together with nine oleasters; cluster 2 (greyish-blue)—includes cultivated varieties mostly from Center Mediterranean; cluster 3 (dark-blue)—almost all cultivated varieties from East Mediterranean; cluster 4, purple—includes 64 out of 73 oleasters (and three cultivated varieties); cluster 5, green—the group corresponding to the 16 samples of subsp. *guanchica*.

When the Bayesian approach was applied, the most probable K was detected in 5 clusters (Figure 2)—the cultivated varieties were split into three sub-populations, represented by clusters 1, 2, 3, respectively, including cultivars from the West, Center, and East Mediterranean; cluster 4 included 64 out of 73 oleasters and only three cultivated varieties; and cluster 4 corresponded to the 16 *guanchica* samples (Table S1), together with the nine remaining oleasters (Figure 2). Allele-frequency divergence among pops (net nucleotide distance), computed using the point estimates of P, showed the highest distances between the oleasters and the cultivated varieties from Central and Eastern Mediterranean (Table S2). The subsp. *guanchica* group displayed similar genetic distance values as the oleasters with the cultivated varieties groups. The lowest value was observed between the oleasters and subsp. *guanchica* samples, followed by the distance between Western and Eastern Mediterranean cultivars. An unexpected high level of admixture was observed in most cases, especially between the cultivated varieties and the oleasters.

Based on these results, which clearly divided the three sources of materials even with few exceptions, the genetic diversity analyses able to individuate possible signs of genes differentiation were performed, which divided the sample set into three groups (cultivar, oleaster, and *guanchica*). 

### 3.2. Genetic Diversity of the Cultivated Varieties, Oleasters, and Subsp. Guanchica Samples

The 1040 EST–SNPs detected on 260 samples were used to evaluate the genetic diversity overall, inside and between each group. The variable polymorphic sites (S) individuated by DnaSP, in the entire sample set, were 637. The standard deviations of heterozygosity, based on the number of variable sites among the sequences (Theta), with and without recombination, were >0.01 and >0.05, respectively. The level of total variance, with or without free recombination, was always lower than 0.01. Nucleotide diversity based on the average pairwise difference among sequences (Pi) was equal to 0.35, while the average number of nucleotide differences (k) was equal to 226.04. When the analysis was applied on each of the three selected groups, subsp. *guanchica*, oleaster, and cultivar, k was equal to 207.21 and Pi was 0.33 for the olive cultivars, while k = 177.67 and 137.95 and Pi equal to 0.28 and 0.22 was detected for the oleasters and subsp. *guanchica*, respectively (Table S3). The Pi results for each group indicated that the cultivated olive maintained a higher level of nucleotide diversity, compared to wild olives and subsp. *guanchica*. When the cultivated samples were matched against the oleaster group, the average number of nucleotide substitution per site (Dxy): 0.405 and number of net nucleotide substitution per site between the cultivated and wild olives (Da): 0.102. Moreover, analyzing the cultivated samples against subsp. *guanchica*, Dxy: 0.400 and Da: 0.129. Finally, for oleasters *versus* subsp. *guanchica*, Dxy: 0.273 and Da: 0.025 (Table 1). The calculated parameters indicated exactly what was previously reported from the above results. In fact, the main differences were between the cultivars and the other two groups, while a higher affinity was found between the oleasters and the subsp. *guanchica*.

The frequency analysis highlighted higher values for the observed heterozygosity than the expected ones for all three analyzed groups, but especially for the olive cultivars (+12%). Moreover, the Fixation Index F, also called the Inbreeding Coefficient, exhibited the lowest and negative value again for the cultivated olive, indicating an excess of heterozygosity, due to negative assortative mating, or a selection for heterozygotes (Table 2). 

Lastly, the Tajima’s D test, applied to determine the allele frequency change, was compared between the wild, cultivated, and subsp. *guanchica* olives. In the cultivated olives, the Tajima’s D values were higher than those in the oleaster and subsp. *guanchica* samples. The Tajima’s value (0.556, *p* > 0.1) indicated that subsp. *guanchica* had a similar variation as expected, which is usually observed in populations growing in equilibrium, without selection evidence. The highest value was detected for the olive cultivars (3.244, *p* < 0.01), two times higher than what was observed for the oleasters. This result might suggest a lack of rare alleles or that the cultivated olives experienced a balancing selection situation or a sudden population contraction.

### 3.3. Identification of Genes Carrying Polymorphisms between Cultivated and Wild Olives

The allele frequencies by population for haploid data (HAFP) analysis was used by merging the two SNP bases in a single data, in order to identify genes with an opposite homozygous state, which were strictly related to each group (Table S4; Figure S1). A total of 124 genes with more than 45% differences between frequencies in each group and an opposite homozygous state were selected. Seventy-two were found between the cultivated and the wild and subsp. *guanchica* groups, 24 between the cultivar and only the oleaster, and 28 between the cultivar and the *guanchica* group.

Several genes were previously annotated in olive, meanwhile, for the genes without any correspondence in olive and for the unknown ones, the amino acid sequences were manually annotated by a BLASTP query, in order to find protein similarities in other plant species. Forty-four genes were not placed in any linkage group of the oleaster genetic map. From the total of 80 localized genes, the highest number of genes in each chromosome were eight genes in chromosome 1 and chromosome 10, seven in chromosome 18 and six in chromosome 12 (Table S5). From all 23 olive chromosomes, none of our detected genes was located in chromosomes 5, 9, and 19. Finally, by using the Expasy translate tool, alleles with different single point mutations were translated into the amino acid sequences and the possible amino acid changes were detected for each allele at each gene. From a total of 124 EST–SNPs, ninety-five were in exons and 21 of them caused amino acid changes, whereas 74 were synonymous SNPs. The other 29 EST-SNPs were located in the UTR regions (Table S5).

The GO functional classification of genes carrying among-group polymorphisms allowed us to verify their involvement in several biological processes (Tables S5 and S6). Most of them were regarding cellular processes (GO:0009987), response to stimulus and defense (GO:0050896; GO:0006952), and metabolic (GO:0008152) and biosynthetic processes (GO:0009058). The 124 genes were located in different cellular components (Table S7), mostly including membrane (GO:0016020), cytosol (GO:0005829), and cell periphery (GO:0071944). Furthermore, several molecular functions were evidenced within the 124 selected contigs (Table S8); binding (GO:0005488; GO:0043167; GO:1901363), catalytic activity (GO:0003824), and transferase activity (GO:0016740) were found.

The specific functions and processes of the 124 genes was also classified. Several genes were related to the abiotic stress response, such as salt stress—gibberellin-regulated protein 14-like GASA14 and nuclear fusion defective 4 NFD4, cold stress: catalase isozyme 3-like CAT3 and 60 S ribosomal protein L23-like, and drought stress—homeobox-leucine zipper protein ATHB-6 and ATHB-12, and aquaporin TIP2-1-like. The resultant gene V-type proton ATPase 16 were linked to cold and drought stresses. Moreover, the gene 30 S ribosomal protein 2 was related to all three abiotic stresses. Genes related to biotic stresses, such as nematode, virus, fungi, and bacteria resistance included the pectate lyase gene family A10 and 18, elongin-C-like, ADP-ribosylation factor-like protein 8a, probable pectinesterase/pectinesterase inhibitor PME34, and fatty acid amide hydrolase-like. Heterogeneous nuclear ribonucleoprotein Q-like was related to fungal and bacterial defense. Finally, cysteine protease RD19A-like was linked to both biotic and abiotic (bacterial, salt and drought) stresses. Seven genes were related to the flowering, five to seed development, whereas the FT-interacting protein 1-like and the two-component response regulator-like PRR37 were related to the photoperiod. Finally, six genes were related to fatty acid synthesis, or involved in the phenolic pathway, such as serine carboxypeptidase-like 7 SCPL and chalcone synthase J-like CHS, or the terpenoid pathway, like geraniol synthase chloroplastic-like GES, geraniol 8-hydroxylase-like, and geraniol 10-hydroxylase G10H.

## 4. Discussion

Plant domestication is an evolutionary process in which humans, through means of artificial selection, take advantage of the genetic diversity of a wild species and modify it, based on their needs [59]. Olive had a peculiar history in its domestication [16,22]. In fact, several cultivated varieties were exported from their original spreading area, located in the eastern side of Mediterranean basin, as seedlings and cuttings, and were diffused by grafting techniques, replacing, and decreasing the olive germplasm diversity of the Central and Western Mediterranean. This scenario played a key role, which contributed to cover and sometimes lose functional polymorphisms private to the western olive germplasm, including oleasters.

The SNP studies performed on olives up to now, were mainly focused on genetic map construction [45,60], genetic diversity studies on restricted set of cultivars [44,46], and few SNP positions were considered on the candidate genes to establish possible phenotypic/genotypic correlations [61,62]. Recently, a large set of SNPs developed on expressed sequence tags was applied for genotyping a wide set of samples, including cultivated and natural forms of olive [47]. The EST-SNP markers, deeply studied in the present work, were gene-based and, therefore, had a higher probability to represent a function-related single nucleotide variant, and were particularly valuable for characterizing genes associated with complex traits [63].

In order to identify genes carrying SNP mutations specific to cultivated and non-cultivated genotypes, in the present study, we deeply analyzed 1040 EST-SNPs on an enriched set from what was previously published [47] of 260 samples, including cultivars, oleasters, and subspecies *guanchica*.

Genetic diversity assessments, based on genetic distance parameters and population structure analyses, confirmed a clear separation between cultivars and oleasters, including trees of the subspecies *guanchica* [12,64,65,66]. The Bayesian analysis revealed that the cultivars were divided into geographically separated populations, including Western, Central, and Eastern Mediterranean groups, as already reported by the SSR genotyping studies [15,16]. Divergence between oleasters and cultivated varieties was higher than that observed among cultivars and the subsp. *guanchica* samples, as observed in the Net nucleotide distance analysis. An upcoming work will elucidate all these differences, especially the differences between the three phylogeographically separated varieties.

The average number of nucleotide differences and nucleotide diversity within each population demonstrated a lower level of nucleotide variation inside the oleasters and subsp. *guanchica* populations, with respect to the cultivated ones. This could also be explained by a lower rate of natural hybridization and introgression because of the narrow geographic environment [63], where the analyzed oleasters and *guanchica* trees were distributed. Conversely, the olive varieties ae cultivated across vast geographical areas [15] and, since they are mostly self-incompatible [67], the hybridization that occurred with the Eastern Mediterranean germplasm with local oleasters and subspecies might have contributed to increasing their variability [13,64,68]. In fact, the average number of differences and the nucleotide diversity were higher in the cultivated materials, with respect to the other groups. These results can reflect from one side, events of hybridization among local genotypes and, from the other, crossing between oleasters spreading in the Western Mediterranean with other olive genotypes coming from the near east [13,15]. The human artificial selection has favored heterozygous genotypes, contributing to the present genetic scenario, as already reported for other plant species [69].

On the entire set of samples, single-point mutations affecting both alleles and distinguishing the cultivated group from the others were deeply analyzed. The EST-SNPs under evaluation were particularly valuable to characterize the genes associated with functional traits [70,71]. Single nucleotide changes might lead to different levels of gene expression or variation on the protein structure, as reported for the non-synonymous as well as synonymous mutations [72,73,74]. Single-point mutations characterizing the three groups were related to 124 genes, and their molecular and biological functions, as well as their cellular component, were reported. Twenty-one EST-SNPs characterizing the cultivated group versus the oleasters and subsp. *guanchica*, generated nsSNPs, leading to amino acid changes. Some EST–SNPs carried by the UTR regions could play a role at the transcription level, or be linked to other functional polymorphisms. The identification of important loci and genes by accessing unique germplasm resources, such as wild populations, might allow their introgression into the cultivated varieties, in order to improve adaptation and tolerance to environmental constrains [75]. The annotation of our candidate genes revealed their involvement in important functional traits, such as stress response, flowering, and secondary metabolite synthesis. An example of genes showing interesting nsSNPs is eRF1, which binds the ribosome A site and recognizes all three stop codons in eukaryotes [76]. When the elongating ribosome encounters a termination codon, this release factor terminates translation and releases the polypeptide from the ribosome, and ribosomal subunits are recycled to participate in a new round of translation [77]. This gene is related to several protein formation and it was recently found to be essential (together with ESP1) in protein-accumulation mechanisms in rice, providing a genetic target for improving relevant agronomic traits [78].

Several genes related to terpenoid, phenol, and fatty acid synthesis were found, such as the fatty acids amide hydrolase (FAAH), where the SNP variation between oleasters and cultivars caused an amino acid change. The FAAH was recently reported to be strictly related to defoliation, mediated by the infection of plants from *Verticillium dahliae*. In fact, the level of FAAH gene expression considerably increased in infected cotton plants [79]. This gene is also well-known for the role in the arachinodic fatty acid synthesis, both in plants and humans, with implications for good health. Glucose-6-Phosphate Transporter 2 (GPT2), whose differential expression in olive under cold stress already showed an nsSNP mutation [80], had a key role in the promotion of cell proliferation, integrated in the growth regulatory network, to enable plant growth and development in continuously changing environmental conditions [81].

Genes related to flowering: One of the selected EST-SNPs was present on the sequence of floricaula/Leafy (LFY), a floral meristem identity gene that plays a key role in flower development [82,83], with a dual function in flower meristem identity regulation and in flowering timing [84]. The LFY promoter contains binding sites for several regulatory factors like Agamous-like MADS-box (AGL) [85], and in Citrus, the constitutive expression of LFY was found to reduce the juvenile phase [86]. An nsSNP was found between wild and cultivated olives in the XTH9 gene, whose expression was reduced in various mutants that had a defect in elongation, including acaulis pin-formed and terminal flower [87]. Moreover, under stress conditions, specific XTH isoforms were up-regulated, while others were down-regulated, suggesting a different involvement of the XTH enzymes on the effects of simultaneous exposure to dehydration and heat conditions, naturally occurring in open fields [88]. An SNP was detected on the 3’UTR region of an FT-interacting protein 1-like. Flowering time in crops represents a critical determinant of the distribution and regional adaptability of plants. In the last decade, knowledge of the florigenic function of Flowering locus T (FT) and FT-like proteins advanced substantially, but the process through which plants control the long-distance action of the FT protein remains largely unknown [89]. The accumulation of FT-like gene transcripts was documented as preceding flower initiation [90]. Expression of OeFT1/2 in olive leaves and OeFT2 in buds was recently studied together, with the expression in transgenic olive cuttings inserting this gene from Arabidopsis, giving very promising results [91]. Two-component response regulator-like gene (PRR37) played a crucial role as a floral repressor, enabling rice to be domesticated and cultivated in high-latitude regions [92]. In olive, this gene is located on chromosome 1 and the single nucleotide change modified the amino acid from aspartate to glutamate. Specific uptake inhibitor (SUI1), which is a protein translation factor, is placed on chromosome 6 in olive, and the nucleotide variation changes the amino acid from arginine to lysine. Overexpression of SUI1 in transgenic rice resulted in an increase in the number of floral organs [93]. Another SNP was detected in the 3’UTR region of the Glycerol-3-phosphate SN-2-acyltransferase 4 gene (GPAT4). A recent examination of pollen and embryo sac, suggested that down-regulation of GPAT4 caused impaired female fertility, which is the main reason for a reduced seed set [94,95]. A synonymous polymorphism was detected within the tetraketide α-pyrone reductase 1-like (TKPR1), a gene involved in the biosynthesis of hydroxylated tetraketide compounds that serve as sporopollenin precursors, the main constituents of pollen coat exine [96]. Another synonymous polymorphism was detected in the 6-phosphogluconate dehydrogenase gene (PGD), described for its important role in the growth of the male gametophytes and pollen-tube–ovule interaction in Arabidopsis [97].

Genes related to stress response: Single nucleotide mutations between wild and cultivated olives generating amino acid changes were detected in two WNK kinase genes (WNK4 and WNK9), respectively, from lysine to arginine (WNK4) and from serine to threonine (WNK9). In plants, the WNK kinase is a large family, with mechanisms to nudge intracellular proteins to their destinations, still to be recognized as a nexus for trafficking-based signal transduction [98]. A synonymous mutation was detected on a hypersensitive-induced response protein-like (HIR). Members of the HIR family are implicated in ion-channel regulation, cell cycle control, and hypersensitive reactions like programmed cell death, in response to pathogen attacks and spontaneous hypersensitive lesions, such as TaHIR3 in wheat and OsHIR1 in rice [99,100]. An SNP change from the apolar glycine to the polar serine in a Pectin Methyl Esterase Inhibitor (PMEI), catalyzes the dimethyl esterification of the homogalacturonan domains of pectin in the plant cell wall, which functions as the primary barrier against pathogens. GhPMEI3 enhances resistance to *Verticillium wilt* in transgenic Arabidopsis [101]. A synonymous mutation was detected on the sequence of abscisic acid receptor (PYL8), required for the ABA-mediated responses, such as stomatal closure and germination inhibition. PYL8 positively regulates ABA signaling during germination and abiotic stress responses. Furthermore, data showed that PYL8 plays essential roles in response to dark-induced senescence [102] and in drought stress [103]. An SNP variation was detected on a Cysteine protease response to dehydration (RD19A-like), which is a responsive gene to salt stress. The cysteine protease genes RD21a and RD19a of *Arabidopsis thaliana* are induced by water deficit and are responsive to salt stress; these play an important role in the programmed cell death pathway during stress [104]. Another SNP was found in a Stress-Related Protein gene (SRPs), found only in higher plants. These lipid droplet (LD) surface proteins are involved in post germinative growth, diurnal regulation of LDs and various stress responses independently or synergistically. However, the cellular mechanism of SRPs in response to abiotic stresses remains unclear. It is possible that SRP-mediated LDs interact with other subcellular organelles, like mitochondria, peroxisomes, and vacuoles under stress conditions [105]. Calcineurin B-like (CBLs) are EF-hand Ca^2+^ protein sensors, and upon Ca^2+^ binding, they undergo conformational changes to associate with a group of CBL-Interacting Protein Kinases (CIPKs). Different combinations of CBLs and CIPKs complexes might generate temporal and spatial specificity in Ca^2+^ signaling, integrating various stimuli to determine cellular responses. CBL could regulate cold-stress-induced signal transduction [106]. The SNP detected on this sequence did not determine any change on the protein sequence. These three latter genes were already tested in a recent trial of salt stress tolerance in olive with very promising results on their different levels of expression under different salt concentration and times (Mousavi et al., under submission). A Probable serine/threonine-protein kinase gene (PBL8), which regulates plant immunity, and might be involved in plant defense signaling [107], holds an inter-population SNP. A mutation distinguished a Protein RNA-directed DNA methylation like (RdDM), an essential factor for suppressing transposons, DNA damage caused by stress, and DNA nucleases. Studies of the RdDM pathway showed that the RDM family proteins are important regulatory factors with various functions that regulate this pathway [108]. A 3’UTR polymorphism was detected on a polyadenylate-binding protein (PABP), involved in salt-tolerance of halophyte plants. This gene plays critical roles in eukaryotic translation initiation and mRNA stabilization/degradation [109]. The Aquaporin-TIP2 is a gene strictly correlated with salt stress and intracellular water transport. The detected SNP is located on chromosome 22 in the olive genome. In tomato, when this is overexpressed, significant increase in fruit yield, harvest index, and plant mass are obtained, and its expression varies in relation to salt concentration in different plant tissues [110]. SlTIP2-2 is a suitable candidate gene to improve important agronomic traits, as well as stress response.

Genes related to metabolic pathways: Two SNPs were found in two different sn-glycerol-3-phosphate acyltransferases (GPAT and GPAT4), a gene family involved in acyl-lipid biosynthesis, triggering storage lipid biosynthesis and catalyzing triacylglycerol (TAG) accumulation, involved in membrane lipid and oil biosynthesis and lipid droplet production in developing pollen grains, which play a pivotal role in plant development [111]. A serine carboxypeptidase -like 7 (SCPL) was placed on chromosome 23, and carried a mutation determining an amino acid change from the apolar glycine to the polar glutamine. The gene was related to phenolic synthesis and it was detected in olive as a gene containing an EST–SSR motif [41]. Phenolic compounds are secondary metabolites involved in several processes, including resistance to biotic and abiotic stresses. The biosynthetic pathways leading to the vast diversity of plant phenolic products often include an acylation step, with phenolic compounds being the donor or acceptor molecules [112]. The biosynthesis of flavonoids from the phenylpropanoid pathway involves the chalcone synthase enzyme. CHS catalyzes the stepwise condensation of three acetate residues from naringenin chalcone, which leads to the synthesis of a variety of flavonoid derivatives [113]. As the first committed enzyme of flavonoid biosynthesis, CHS is rigorously controlled in response to a wide range of environmental and developmental stimuli. Flavonoids also function as antimicrobial compounds (phytoalexins) or insect repellents defending against phytopathogens and herbivores, and some flavonoid metabolites have pharmacological activities [114]. A chalcone synthase gene was already sequenced in olive, revealing a total of nine putative SNPs [115]. In the present study, a single sSNP was detected. An SNP variation was in charge of a Geraniol 10-hydroxylase (G10H), thought to play an important role in iridoid monoterpenoid biosynthesis. Recently, it was shown that the CrDXS and CrG10H genes isolated from *Catharanthus roseus* are important genes in regulating terpenoid indole alkaloid biosynthesis, filling knowledge gaps in understanding the pathway [116,117,118,119]. A 3’UTR SNP was located on a biotin carboxyl carrier protein of acetyl-CoA carboxylase 1 (BCCP), the first committed step in fatty acid synthesis, mediated by acetyl-CoA carboxylase (ACCase), a biotin-dependent enzyme that carboxylates acetyl-CoA to produce malonyl-CoA. The heteromeric ACCase is composed of four distinct subunits—biotin carboxyl transfer protein (BCCP), biotin carboxylase (BC), and a/b-carboxyltransferases (CT). These subunits are organized in two separable subcomplexes, one comprising BCCP and BC, which mediates the carboxylation of biotin, and a second a-CT/b-CT complex that catalyzes the carboxyltransferase reaction [120,121]. Olive produces a range of secondary metabolites that strongly affect the taste and nutritional properties of olive oil and fruits. Among them, the most abundant are the secoiridoids and monoterpenoids with a 3,4-dihydropyran skeleton. Some amino acid substitutions were observed in the conserved motifs of the olive 1,4-reductase [117], but the SNP identified in this work did not produce any amino acid change.

Among the EST-SNPs distinguishing cultivated and wild olive populations, 21 determining amino acid changes of enzymes directly affecting plant performance, represent an important step towards the identification of useful markers for the functional screening of olive resources or sequences useful for genetic improvement by classical breeding or via cisgenesis.

## 5. Conclusions

Tracing the selection footprint in combination with the identification of causative variations, would inform the future detection of important loci correlated to plant behavior and cultivation. The identification of markers specific to cultivated or wild olives might represent a new resource for the introgression of important variation into the cultivated olives through newly developed biotechniques, such as cisgenesis. The present study is an evidence of the importance of safeguarding the genetic diversity of the *Olea europaea* species. In fact, the availability in situ and ex situ of wild plants, together with the knowledge of variation carried by oleasters will allow the restoration or establishment of new allelic combinations, giving new opportunities for improving adaptation, stress response, or modifying the main metabolic pathways in cultivated olive, also to overcome future scenarios of climate change.

## Figures and Tables

**Table 1:** Genetic differentiation parameters between cultivars, oleasters, and subsp. *guanchica* samples detected by DnaSP 6.

Group 1	Group 2	*Ks*	*Kxy*	Gst	DeltaSt	GammaSt	Nst	Fst	Dxy	Da
Cultivars	Wild olives	19.837 ***	25.770 ***	0.000	0.043	0.123	0.330	0.253	0.405	0.102
Cultivars	subsp. *guanchica*	20.128 ***	25.492 ***	0.006	0.020	0.062	0.402	0.323	0.400	0.129
Wild olives	subsp. *guanchica*	17.052 ***	17.386 ***	0.004	0.008	0.032	0.107	0.092	0.273	0.025

*Ks* and *Kxy—*genetic differentiation parameters by the PM test; probability obtained by the permutation test with 1000 replicates, *** *p* < 0.001. Gst—Gene flow parameters, based on haplotype data information; DeltaSt, GammaSt, Nst, and Fst, based on sequence data information. Dxy—average number of nucleotide substitutions per site; Da—number of net nucleotide substitutions per site.

**Table 2:** Heterozygosity, F-statistics, and polymorphism by population.

Group		N	Na	Ne	Ho	He	UHe	F
Cultivar	Mean	170.43	2.013	1.536	0.369	0.324	0.325	−0.120
	SE	0.031	0.004	0.009	0.005	0.004	0.004	0.005
var. *sylvestris*	Mean	72.92	1.976	1.448	0.295	0.275	0.276	−0.055
	SE	0.011	0.005	0.010	0.006	0.005	0.005	0.006
subsp. *guanchica*	Mean	15.92	1.738	1.345	0.214	0.206	0.212	−0.027
	SE	0.012	0.014	0.011	0.007	0.006	0.006	0.009
Total	Mean	86.43	1.909	1.443	0.293	0.268	0.271	−0.071
	SE	1.142	0.005	0.006	0.004	0.003	0.003	0.004

SE—Standard Error; N—Sample Size; Na—number of alleles; Ne—effective alleles; Ho—observed heterozygosity; He—expected heterozygosity, UHe—unbiased expected heterozygosity; and F—Fixation Index.

## Supplementary Materials

The following are available online at https://www.mdpi.com/2073-4425/11/8/916/s1, Figure S1: Genes carrying SNP polymorphisms between cultivated, oleaster, and subsp. *guanchica* olives. Table S1. List of olive samples analyzed, country/place of origin/diffusion. Table S2: Allele-frequency divergence among populations (Net nucleotide distance), computed using point estimates of P, using the Structure software. EM, WM, and CM, respectively, refer to East, West, and Central Mediterranean cultivars. Table S3: Genetic diversity for each population and the overall sample set, performed by the DnaSP software. Table S4: Haploid Allele Frequencies and Sample Size by Population differences in homozygosity state. Blue loci: Cultivated olives versus oleasters and subp. *Guanchica*. iPurple loci: Cultivars versus oleasters. Green loci: Cultivars versus subsp. *Guanchica*. Table S5: List of the 124 genes differentiating olive cultivars from oleasters and subsp. *guanchica* samples. Name of contigs, linkage group placement, NCBI accession number, gene name, and molecular process and function for each gene are reported. Table S6: List of the biological processes detected for the 124 genes differentiating olive cultivars from oleasters and subsp. *guanchica* samples. The analysis was performed by the PANTHER Overrepresentation Test. Table S7: List of all cellular components detected for the 124 genes differentiating olive cultivars from the oleasters and subsp. *guanchica* samples. The analysis was performed by the PANTHER Overrepresentation Test. Table S8: List of all molecular functions detected for the 124 genes differentiating olive cultivars from the oleasters and subsp. *guanchica* samples. The analysis was performed by the PANTHER Overrepresentation Test.
