# Supplementary material for: EST–SNP Study of Olea europaea L. Uncovers Functional Polymorphisms between Cultivated and Wild Olives
Source: Genes (Basel). 2020 Aug 10;11(8):916. doi: 10.3390/genes11080916 (PMC7465833; doi:10.3390/genes11080916)
Supplement: Supplementary file 1 [file genes-11-00916-s001.zip › Table_S1.docx]

**Table S1.** List of olive samples analyzed, country/place of origin/diffusion.

| **Olive cultivars** (*Olea europaea* subp. *europaea* var. *europaea*) | **Country of origin/diffusion** |
| --- | --- |
| Klon | Albania |
| Mixani | Albania |
| Ulliri I Kuq | Albania |
| Chemlal de Kaby~~i~~li~~y~~e | Algeria |
| Arauco | Argentina |
| Kato Drys | Cyprus |
| Kiti | Cyprus |
| Toffahi 1000 | Egypt |
| Lucques | France |
| Oliviere | France |
| Picholine* | France |
| Chalkidikis | Greece |
| Kalamon* | Greece |
| Kerkiras | Greece |
| Koroneiki | Greece |
| Mastoidis | Greece |
| Maureya | Greece |
| Myrtolia | Greece |
| Valanolia | Greece |
| Fishomi** | Iran |
| Mari** | Iran |
| Ordib** | Iran |
| Shengeh** | Iran |
| Barnea | Israel |
| Abunara | Italy |
| Americano | Italy |
| Ascolana Tenera* | Italy |
| Bianchera* | Italy |
| Biancolilla | Italy |
| Borgiona* | Italy |
| Bosana* | Italy |
| Calatina | Italy |
| Canino* | Italy |
| Carolea* | Italy |
| Cavalieri | Italy |
| Cerasuola* | Italy |
| Cima di Melfi | Italy |
| Cipressino | Italy |
| Coratina* | Italy |
| Craputea | Italy |
| Crastu | Italy |
| Cuoricino | Italy |
| Dolce Agogia* | Italy |
| Faresana | Italy |
| Fasolina | Italy |
| Fasolona | Italy |
| Frantoio* | Italy |
| Gargnà~~a~~ | Italy |
| Gentile di Chieti* | Italy |
| Grappolo | Italy |
| Grossa di Spagna | Italy |
| Intosso | Italy |
| Itrana* | Italy |
| Leccino* | Italy |
| Leccio Maremmano | Italy |
| Lumiaro | Italy |
| Maiatica di Ferrandina | Italy |
| Majorca | Italy |
| Maurino* | Italy |
| Moraiolo* | Italy |
| Morchiaio | Italy |
| Moresca | Italy |
| Nasitana | Italy |
| Nocellara del Belice | Italy |
| Nocellara Etnea | Italy |
| Nostrale di Rigali* | Italy |
| Nostrana di Brisighella* | Italy |
| Ogliarola del Bradano | Italy |
| Ogliarola del Vulture | Italy |
| Olivastra Seggianese | Italy |
| Olivo di Mandanici | Italy |
| Passalunara* | Italy |
| Pendolino | Italy |
| Piantone di Mogliano* | Italy |
| Pidicuddara | Italy |
| Pisciottana | Italy |
| Raio* | Italy |
| Ravece | Italy |
| Rossellino | Italy |
| Rossello | Italy |
| Salicino | Italy |
| San Francesco | Italy |
| Sant’ Agostino | Italy |
| Santa Martinenga | Italy |
| Selvatico | Italy |
| Semidana | Italy |
| Sivigliana da Olio | Italy |
| Tonda Iblea* | Italy |
| Uovo di Piccione | Italy |
| Verdella | Italy |
| Beladi | Lebanon |
| Picholine Mar~~r~~ocaine | Morocco |
| Alameno de Cabra | Spain |
| Arbequina | Spain |
| Arbosana | Spain |
| Blanqueta | Spain |
| Bolvino | Spain |
| Canetera | Spain |
| Canivano Negro | Spain |
| Carrasqueñ~~n~~o de Elvas | Spain |
| Carrasqueñ~~n~~o de Porcuna | Spain |
| Cerezuela | Spain |
| Changlot Real | Spain |
| Corbella | Spain |
| Empeltre | Spain |
| Figueretes | Spain |
| Forastera de Tortosa | Spain |
| Gatuno | Spain |
| Gordal Sevillana | Spain |
| Grosal de Cieza | Spain |
| Hojiblanca | Spain |
| Jabaluna | Spain |
| Joanenca | Spain |
| Lechin de Sevilla | Spain |
| Lentisca | Spain |
| Llumeta | Spain |
| Manzanilla Cacereñ~~n~~a | Spain |
| Manzanilla de Almeria | Spain |
| Manzanilla de Ayora | Spain |
| Manzanilla de Sevilla | Spain |
| Manzanillera de Huercal Overa | Spain |
| Manzanillo de Santisteban | Spain |
| Menya | Spain |
| Mollar de Cieza | Spain |
| Morrut | Spain |
| Nevadillo Blanco de Lucena | Spain |
| Ocal 25 | Spain |
| Ocal 427 | Spain |
| Palomar | Spain |
| Pico Limon | Spain |
| Picual | Spain |
| Picudo | Spain |
| Piñ~~n~~onera | Spain |
| Redondilla de Logrono | Spain |
| Temprano | Spain |
| Verdial de Badajoz | Spain |
| Verdial de Velez | Spain |
| Verdial de Velez Malaga | Spain |
| Zarzariega de Orcera | Spain |
| Abbadi | Syria |
| Abbadi Abou | Syria |
| Abbadi Abou Gabra | Syria |
| Abou Kanani | Syria |
| Abou Satl Mohazam | Syria |
| Adkam | Syria |
| Barri | Syria |
| Doebli | Syria |
| Jabali | Syria |
| Kelb et Ter | Syria |
| Khalkhali | Syria |
| Khashabi | Syria |
| Maarri | Syria |
| Mahati | Syria |
| Majhol 1013 | Syria |
| Majhol 152 | Syria |
| Massabi | Syria |
| Safrawi | Syria |
| Shami | Syria |
| Toffahi 721 | Syria |
| Zaity | Syria |
| Chemlali* | Tunisia |
| Chetoui* | Tunisia |
| Marsaline | Tunisia |
| Ayvalik | Turkey |
| Dokkar | Turkey |
| Domat* | Turkey |
| Gemlik | Turkey |
| Izmir Sofralik* | Turkey |
| Kiraz | Turkey |
| Uslu | Turkey |
| Yun Celebi | Turkey |
| **Wild olives** (*Olea europaea* subp. *europaea* var. *sylvestris*) | **Region/Place of origin***** |
| W1 | Andalusia/Cadiz |
| W2 | Andalusia/Cadiz |
| W3 | Andalusia/Cadiz |
| W7 | Andalusia/Cadiz |
| W20 | Andalusia/Cadiz |
| W24 | Andalusia/Cadiz |
| W29 | Andalusia/Cadiz |
| W44 | Andalusia/Cadiz |
| W45 | Andalusia/Cadiz |
| W65 | Andalusia/Cadiz |
| W70 | Andalusia/Cadiz |
| W71 | Andalusia/Cadiz |
| W72 | Andalusia/Cadiz |
| W157 | Andalusia/Cadiz |
| W190 | Andalusia/Cadiz |
| W8 | Andalusia/Jaen |
| W10 | Andalusia/Jaen |
| W17 | Andalusia/Jaen |
| W19 | Andalusia/Jaen |
| W40 | Andalusia/Jaen |
| W51 | Andalusia/Jaen |
| W74 | Andalusia/Jaen |
| W75 | Andalusia/Jaen |
| W132 | Andalusia/Jaen |
| W133 | Andalusia/Jaen |
| W53 | Andalusia/Seville |
| W54 | Andalusia/Seville |
| W55 | Andalusia/Seville |
| W56 | Andalusia/Seville |
| W57 | Andalusia/Seville |
| W58 | Andalusia/Seville |
| W59 | Andalusia/Seville |
| W60 | Andalusia/Seville |
| W61 | Andalusia/Seville |
| W62 | Andalusia/Seville |
| W163 | Extremadura |
| W164 | Extremadura |
| W165 | Extremadura |
| W166 | Extremadura |
| W167 | Extremadura |
| W168 | Extremadura |
| W170 | Extremadura |
| W171 | Extremadura |
| W172 | Extremadura |
| W173 | Extremadura |
| W174 | Extremadura |
| W175 | Extremadura |
| W176 | Extremadura |
| W177 | Extremadura |
| W178 | Extremadura |
| W179 | Extremadura |
| W180 | Extremadura |
| W181 | Extremadura |
| W182 | Extremadura |
| W183 | Extremadura |
| W184 | Extremadura |
| W26 | Balearic Islands/Mallorca |
| W27 | Balearic Islands/Mallorca |
| W28 | Balearic Islands/Mallorca |
| W64 | Balearic Islands/Mallorca |
| W90 | Balearic Islands/Mallorca |
| W91 | Balearic Islands/Mallorca |
| W93 | Balearic Islands/Mallorca |
| W96 | Balearic Islands/Mallorca |
| W97 | Balearic Islands/Mallorca |
| W78 | Balearic Islands/Menorca |
| W79 | Balearic Islands/Menorca |
| W81 | Balearic Islands/Menorca |
| W82 | Balearic Islands/Menorca |
| W84 | Balearic Islands/Menorca |
| W86 | Balearic Islands/Menorca |
| W88 | Balearic Islands/Menorca |
| W113 | Balearic Islands/Menorca |
| ***guanchica* samples** (*Olea europaea* subp. *guanchica*) | **Region/Place of origin***** |
| subsp. *guanchica* 25 | Canary Islands/Gran Canarias |
| subsp. *guanchica* 46 | Canary Islands/Gran Canarias |
| subsp. *guanchica* 47 | Canary Islands/Gran Canarias |
| subsp. *guanchica* 118 | Canary Islands/Gran Canarias |
| subsp. *guanchica* 120 | Canary Islands/Gran Canarias |
| subsp. *guanchica* 121 | Canary Islands/Gran Canarias |
| subsp. *guanchica* 135 | Canary Islands/Gran Canarias |
| subsp. *guanchica* 137 | Canary Islands/Gran Canarias |
| subsp. *guanchica* 32 | Canary Islands/Tenerife |
| subsp. *guanchica* 33 | Canary Islands/Tenerife |
| subsp. *guanchica* 35 | Canary Islands/Tenerife |
| subsp. *guanchica* 36 | Canary Islands/Tenerife |
| subsp. *guanchica* 48 | Canary Islands/Tenerife |
| subsp. *guanchica* 49 | Canary Islands/Tenerife |
| subsp. *guanchica* 130 | Canary Islands/Tenerife |
| subsp. *guanchica* 297 | Canary Islands/La Gomera |

All samples derive from the WOGB olive collection of Cordoba, Spain (Belaj et al., 2018).

*Samples from the International Olive Collection of Lugnano (Terni,Italy).

**from DNA repository of CNR-IBBR of Perugia.

*** All wild olives and subsp. *guanchica* samples were collected from Spain including Canary and Balearic Islands.

**Reference**

Belaj, A.; De La Rosa, R.; Lorite, I.J.; Mariotti, R.; Cultrera, N.G.; Beuzón, C.R.; González-Plaza, J.J.; Muñoz-Mérida, A.; Trelles, O.; Baldoni, L. Usefulness of a New Large Set of High Throughput EST-SNP Markers as a Tool for Olive Germplasm Collection Management. Front. Plant Sci. **2018**, 9.
