# Supplementary material for: EST–SNP Study of Olea europaea L. Uncovers Functional Polymorphisms between Cultivated and Wild Olives
Source: Genes (Basel). 2020 Aug 10;11(8):916. doi: 10.3390/genes11080916 (PMC7465833; doi:10.3390/genes11080916)
Supplement: Supplementary file 1 [file genes-11-00916-s001.zip › Table_S2.docx]

**Table S2.** Allele-frequency divergence among populations (Net nucleotide distance), computed using point estimates of P, by Structure software. EM, WM and CM respectively refer to East, West and Central Mediterranean cultivars.

| **Pops** | **EM Cultivars** | **WM Cultivars** | **CM Cultivars** | **Wild olives** | ***guanchica* samples** |
| --- | --- | --- | --- | --- | --- |
| **EM Cultivars** | - |  |  |  |  |
| **WM Cultivars** | 0.0543 | - |  |  |  |
| **CM Cultivars** | 0.0578 | 0.0722 | - |  |  |
| **Wild olives** | 0.2426 | 0.2242 | 0.2563 | - |  |
| ***guanchica* samples** | 0.1921 | 0.1833 | 0.2111 | 0.0519 | - |
