# Supplementary material for: EST–SNP Study of Olea europaea L. Uncovers Functional Polymorphisms between Cultivated and Wild Olives
Source: Genes (Basel). 2020 Aug 10;11(8):916. doi: 10.3390/genes11080916 (PMC7465833; doi:10.3390/genes11080916)
Supplement: Supplementary file 1 [file genes-11-00916-s001.zip › Table_S3.docx]

**Table S3.** Genetic diversity, for each population and overall sample set, performed by DnaSP software.

| **Population** | **h** | **S** | **K** | **Pi** | **PiJC** |
| --- | --- | --- | --- | --- | --- |
| Cultivars | 342 | 637 | 207.21 | 0.33 | 0.43 |
| Wils types | 146 | 625 | 177.67 | 0.28 | 0.36 |
| Subp. *guanchica* | 32 | 474 | 137.95 | 0.22 | 0.26 |
| Total | 520 | 637 | 226.04 | 0.35 | - |

h: number of haplotypes; S: number of segregating sites; K: average number of differences; Pi: nucleotide diversity; PiJC: nucleotide diversity by using Jukes and Cantor correction
