# Supplementary material for: EST–SNP Study of Olea europaea L. Uncovers Functional Polymorphisms between Cultivated and Wild Olives
Source: Genes (Basel). 2020 Aug 10;11(8):916. doi: 10.3390/genes11080916 (PMC7465833; doi:10.3390/genes11080916)
Supplement: Supplementary file 1 [file genes-11-00916-s001.zip › Table_S4.docx]

**Table S4.** Haploid Allele Frequencies and Sample Size by Population differences in homozygosity state. Blue loci: cultivated versus wild olives and subp. *guanchica*; iPurple loci: cultivars versus wilds; Green loci: cultivars versus subsp. *guanchica*.

| **Locus** | **Allele** | **Cultivars** | **Wild olives** | **subsp. *guanchica*** | **Frequency differences among groups** | | |
| --- | --- | --- | --- | --- | --- | --- | --- |
|  |  |  |  |  | ***versus* wilds** | | ***versus* subp. *guanchica*** |
| **10026** | **N** | 171 | 73 | 16 |  |  | |
|  | **TT** | 0.713 | 0.068 | 0.063 | 64% | 65% | |
|  | **CC** | 0.000 | 0.534 | 0.625 | -53% | -63% | |
| **10109** | **N** | 171 | 73 | 16 |  |  | |
|  | **AA** | 0.006 | 0.493 | 0.875 | -49% | -87% | |
|  | **GG** | 0.737 | 0.110 | 0.000 | 63% | 74% | |
| **10266** | **N** | 171 | 73 | 16 |  |  | |
|  | **TT** | 0.094 | 0.548 | 0.625 | -45% | -53% | |
|  | **CC** | 0.526 | 0.055 | 0.000 | 47% | 53% | |
| **10405** | **N** | 170 | 72 | 16 |  |  | |
|  | **TT** | 0.629 | 0.000 | 0.000 | 63% | 63% | |
|  | **CC** | 0.006 | 0.722 | 0.938 | -72% | -93% | |
| **10411** | **N** | 171 | 73 | 16 |  |  | |
|  | **AA** | 0.035 | 0.658 | 0.938 | -62% | -90% | |
|  | **GG** | 0.637 | 0.055 | 0.000 | 58% | 64% | |
| **10481** | **N** | 171 | 73 | 16 |  |  | |
|  | **TT** | 0.006 | 0.575 | 0.938 | -57% | -93% | |
|  | **CC** | 0.661 | 0.082 | 0.000 | 58% | 66% | |
| **1064** | **N** | 168 | 73 | 16 |  |  | |
|  | **AA** | 0.024 | 0.493 | 0.938 | -47% | -91% | |
|  | **GG** | 0.613 | 0.082 | 0.000 | 53% | 61% | |
| **10672** | **N** | 171 | 73 | 16 |  |  | |
|  | **AA** | 0.012 | 0.712 | 1.000 | -70% | -99% | |
|  | **TT** | 0.561 | 0.041 | 0.000 | 52% | 56% | |
| **11058** | **N** | 171 | 73 | 16 |  |  | |
|  | **AA** | 0.041 | 0.712 | 0.875 | -67% | -83% | |
|  | **GG** | 0.579 | 0.027 | 0.000 | 55% | 58% | |
| **11296** | **N** | 171 | 73 | 16 |  |  | |
|  | **TT** | 0.655 | 0.055 | 0.063 | 60% | 59% | |
|  | **CC** | 0.012 | 0.616 | 0.875 | -60% | -86% | |
| **11541_2** | **N** | 171 | 73 | 16 |  |  | |
|  | **TT** | 0.713 | 0.014 | 0.000 | 70% | 71% | |
|  | **GG** | 0.006 | 0.616 | 0.688 | -61% | -68% | |
| **11650** | **N** | 170 | 73 | 16 |  |  | |
|  | **AA** | 0.688 | 0.041 | 0.000 | 65% | 69% | |
|  | **GG** | 0.012 | 0.644 | 0.875 | -63% | -86% | |
| **11682** | **N** | 171 | 73 | 16 |  |  | |
|  | **TT** | 0.661 | 0.041 | 0.000 | 62% | 66% | |
|  | **CC** | 0.047 | 0.644 | 0.938 | -60% | -89% | |
| **11788** | **N** | 171 | 71 | 16 |  |  | |
|  | **TT** | 0.784 | 0.169 | 0.000 | 61% | 78% | |
|  | **CC** | 0.012 | 0.521 | 0.938 | -51% | -93% | |
| **12110** | **N** | 169 | 73 | 16 |  |  | |
|  | **AA** | 0.639 | 0.000 | 0.063 | 64% | 58% | |
|  | **TT** | 0.012 | 0.644 | 0.875 | -63% | -86% | |
| **135** | **N** | 171 | 73 | 16 |  |  | |
|  | **AA** | 0.713 | 0.027 | 0.000 | 69% | 71% | |
|  | **GG** | 0.047 | 0.644 | 0.938 | -60% | -89% | |
| **1395** | **N** | 171 | 73 | 16 |  |  | |
|  | **TT** | 0.070 | 0.712 | 0.938 | -64% | -87% | |
|  | **CC** | 0.497 | 0.041 | 0.000 | 46% | 50% | |
| **1650** | **N** | 168 | 73 | 16 |  |  | |
|  | **TT** | 0.030 | 0.699 | 0.875 | -67% | -85% | |
|  | **GG** | 0.506 | 0.014 | 0.000 | 49% | 51% | |
| **1684** | **N** | 171 | 73 | 16 |  |  | |
|  | **AA** | 0.006 | 0.671 | 0.875 | -67% | -87% | |
|  | **GG** | 0.614 | 0.027 | 0.000 | 59% | 61% | |
| **1775** | **N** | 171 | 73 | 16 |  |  | |
|  | **TT** | 0.503 | 0.041 | 0.000 | 46% | 50% | |
|  | **CC** | 0.041 | 0.726 | 0.875 | -69% | -83% | |
| **2011** | **N** | 171 | 73 | 16 |  |  | |
|  | **AA** | 0.018 | 0.630 | 0.938 | -61% | -92% | |
|  | **GG** | 0.591 | 0.014 | 0.000 | 58% | 59% | |
| **2040** | **N** | 170 | 73 | 16 |  |  | |
|  | **TT** | 0.018 | 0.521 | 0.688 | -50% | -67% | |
|  | **CC** | 0.618 | 0.137 | 0.000 | 48% | 62% | |
| **208** | **N** | 171 | 73 | 16 |  |  | |
|  | **TT** | 0.655 | 0.014 | 0.063 | 64% | 59% | |
|  | **CC** | 0.012 | 0.726 | 0.813 | -71% | -80% | |
| **2104** | **N** | 171 | 73 | 16 |  |  | |
|  | **AA** | 0.509 | 0.014 | 0.000 | 50% | 51% | |
|  | **GG** | 0.058 | 0.603 | 0.875 | -54% | -82% | |
| **2159** | **N** | 170 | 73 | 16 |  |  | |
|  | **TT** | 0.006 | 0.534 | 0.875 | -53% | -87% | |
|  | **CC** | 0.753 | 0.041 | 0.000 | 71% | 75% | |
| **2331** | **N** | 169 | 73 | 16 |  |  | |
|  | **TT** | 0.751 | 0.041 | 0.000 | 71% | 75% | |
|  | **CC** | 0.012 | 0.644 | 0.875 | -63% | -86% | |
| **2607** | **N** | 171 | 73 | 16 |  |  | |
|  | **TT** | 0.012 | 0.521 | 0.875 | -51% | -86% | |
|  | **GG** | 0.667 | 0.055 | 0.000 | 61% | 67% | |
| **2690** | **N** | 171 | 73 | 16 |  |  | |
|  | **AA** | 0.602 | 0.000 | 0.000 | 60% | 60% | |
|  | **GG** | 0.012 | 0.767 | 0.938 | -76% | -93% | |
| **2724** | **N** | 170 | 73 | 16 |  |  | |
|  | **TT** | 0.612 | 0.055 | 0.000 | 56% | 61% | |
|  | **CC** | 0.041 | 0.658 | 0.938 | -62% | -90% | |
| **2748** | **N** | 171 | 73 | 16 |  |  | |
|  | **TT** | 0.766 | 0.041 | 0.000 | 72% | 77% | |
|  | **CC** | 0.018 | 0.658 | 0.875 | -64% | -86% | |
| **2824** | **N** | 170 | 73 | 16 |  |  | |
|  | **AA** | 0.024 | 0.575 | 0.813 | -55% | -79% | |
|  | **GG** | 0.629 | 0.041 | 0.063 | 59% | 57% | |
| **288** | **N** | 170 | 73 | 16 |  |  | |
|  | **TT** | 0.006 | 0.589 | 0.938 | -58% | -93% | |
|  | **CC** | 0.782 | 0.082 | 0.000 | 70% | 78% | |
| **3022** | **N** | 171 | 73 | 16 |  |  | |
|  | **TT** | 0.018 | 0.630 | 0.875 | -61% | -86% | |
|  | **CC** | 0.754 | 0.027 | 0.000 | 73% | 75% | |
| **3158** | **N** | 171 | 73 | 16 |  |  | |
|  | **TT** | 0.643 | 0.082 | 0.000 | 56% | 64% | |
|  | **CC** | 0.000 | 0.521 | 1.000 | -52% | -100% | |
| **319** | **N** | 170 | 73 | 16 |  |  | |
|  | **TT** | 0.024 | 0.589 | 0.938 | -57% | -91% | |
|  | **CC** | 0.706 | 0.151 | 0.000 | 56% | 71% | |
| **3236** | **N** | 171 | 73 | 16 |  |  | |
|  | **AA** | 0.070 | 0.630 | 0.813 | -56% | -74% | |
|  | **GG** | 0.497 | 0.027 | 0.000 | 47% | 50% | |
| **344** | **N** | 171 | 73 | 16 |  |  | |
|  | **TT** | 0.018 | 0.630 | 0.875 | -61% | -86% | |
|  | **CC** | 0.596 | 0.027 | 0.000 | 57% | 60% | |
| **3481** | **N** | 171 | 73 | 16 |  |  | |
|  | **AA** | 0.591 | 0.055 | 0.000 | 54% | 59% | |
|  | **GG** | 0.064 | 0.589 | 0.875 | -52% | -81% | |
| **3549** | **N** | 171 | 73 | 16 |  |  | |
|  | **TT** | 0.006 | 0.575 | 0.813 | -57% | -81% | |
|  | **CC** | 0.673 | 0.041 | 0.000 | 63% | 67% | |
| **3597** | **N** | 171 | 73 | 16 |  |  | |
|  | **AA** | 0.708 | 0.055 | 0.188 | 65% | 52% | |
|  | **GG** | 0.012 | 0.575 | 0.563 | -56% | -55% | |
| **3601** | **N** | 171 | 73 | 16 |  |  | |
|  | **AA** | 0.047 | 0.644 | 0.938 | -60% | -89% | |
|  | **GG** | 0.591 | 0.027 | 0.000 | 56% | 59% | |
| **3710** | **N** | 167 | 73 | 16 |  |  | |
|  | **AA** | 0.006 | 0.589 | 0.938 | -58% | -93% | |
|  | **GG** | 0.605 | 0.041 | 0.000 | 56% | 60% | |
| **4188** | **N** | 171 | 73 | 16 |  |  | |
|  | **AA** | 0.082 | 0.781 | 1.000 | -70% | -92% | |
|  | **CC** | 0.485 | 0.027 | 0.000 | 46% | 49% | |
| **4374** | **N** | 171 | 72 | 16 |  |  | |
|  | **TT** | 0.614 | 0.056 | 0.000 | 56% | 61% | |
|  | **CC** | 0.012 | 0.667 | 0.688 | -65% | -68% | |
| **4375** | **N** | 171 | 73 | 16 |  |  | |
|  | **AA** | 0.678 | 0.055 | 0.000 | 62% | 68% | |
|  | **GG** | 0.000 | 0.562 | 0.875 | -56% | -88% | |
| **4534** | **N** | 170 | 73 | 16 |  |  | |
|  | **AA** | 0.688 | 0.082 | 0.188 | 61% | 50% | |
|  | **GG** | 0.012 | 0.575 | 0.500 | -56% | -49% | |
| **4599** | **N** | 171 | 73 | 16 |  |  | |
|  | **AA** | 0.000 | 0.521 | 0.500 | -52% | -50% | |
|  | **CC** | 0.807 | 0.068 | 0.125 | 74% | 68% | |
| **4714** | **N** | 171 | 73 | 16 |  |  | |
|  | **AA** | 0.018 | 0.630 | 0.875 | -61% | -86% | |
|  | **GG** | 0.731 | 0.041 | 0.000 | 69% | 73% | |
| **4794** | **N** | 171 | 73 | 16 |  |  | |
|  | **AA** | 0.041 | 0.781 | 1.000 | -74% | -96% | |
|  | **CC** | 0.509 | 0.027 | 0.000 | 48% | 51% | |
| **4851** | **N** | 171 | 73 | 16 |  |  | |
|  | **AA** | 0.006 | 0.685 | 0.875 | -68% | -87% | |
|  | **GG** | 0.608 | 0.027 | 0.063 | 58% | 55% | |
| **504** | **N** | 171 | 73 | 16 |  |  | |
|  | **AA** | 0.596 | 0.014 | 0.000 | 58% | 60% | |
|  | **CC** | 0.006 | 0.671 | 0.938 | -67% | -93% | |
| **5193** | **N** | 171 | 73 | 16 |  |  | |
|  | **TT** | 0.018 | 0.603 | 0.938 | -59% | -92% | |
|  | **CC** | 0.596 | 0.137 | 0.000 | 46% | 60% | |
| **5398** | **N** | 168 | 73 | 16 |  |  | |
|  | **TT** | 0.518 | 0.041 | 0.000 | 48% | 52% | |
|  | **CC** | 0.048 | 0.534 | 0.938 | -49% | -89% | |
| **5822** | **N** | 171 | 73 | 16 |  |  | |
|  | **AA** | 0.626 | 0.014 | 0.000 | 61% | 63% | |
|  | **GG** | 0.000 | 0.658 | 0.750 | -66% | -75% | |
| **6061** | **N** | 170 | 73 | 16 |  |  | |
|  | **TT** | 0.653 | 0.068 | 0.000 | 58% | 65% | |
|  | **CC** | 0.024 | 0.671 | 0.875 | -65% | -85% | |
| **6453** | **N** | 171 | 73 | 16 |  |  | |
|  | **AA** | 0.509 | 0.014 | 0.000 | 50% | 51% | |
|  | **GG** | 0.064 | 0.808 | 0.875 | -74% | -81% | |
| **6604** | **N** | 170 | 73 | 16 |  |  | |
|  | **TT** | 0.018 | 0.630 | 0.875 | -61% | -86% | |
|  | **CC** | 0.682 | 0.041 | 0.000 | 64% | 68% | |
| **662** | **N** | 171 | 73 | 16 |  |  | |
|  | **TT** | 0.006 | 0.712 | 1.000 | -71% | -99% | |
|  | **CC** | 0.649 | 0.068 | 0.000 | 58% | 65% | |
| **686** | **N** | 171 | 73 | 16 |  |  | |
|  | **AA** | 0.637 | 0.027 | 0.000 | 61% | 64% | |
|  | **GG** | 0.000 | 0.685 | 0.750 | -68% | -75% | |
| **6913** | **N** | 171 | 73 | 16 |  |  | |
|  | **TT** | 0.509 | 0.027 | 0.000 | 48% | 51% | |
|  | **CC** | 0.082 | 0.575 | 0.813 | -49% | -73% | |
| **6929** | **N** | 170 | 73 | 16 |  |  | |
|  | **TT** | 0.006 | 0.630 | 0.938 | -62% | -93% | |
|  | **CC** | 0.706 | 0.082 | 0.000 | 62% | 71% | |
| **722** | **N** | 171 | 73 | 16 |  |  | |
|  | **AA** | 0.778 | 0.082 | 0.000 | 70% | 78% | |
|  | **TT** | 0.012 | 0.548 | 0.938 | -54% | -93% | |
| **7348** | **N** | 171 | 71 | 16 |  |  | |
|  | **TT** | 0.012 | 0.577 | 0.938 | -57% | -93% | |
|  | **CC** | 0.620 | 0.085 | 0.063 | 54% | 56% | |
| **7809** | **N** | 171 | 73 | 16 |  |  | |
|  | **TT** | 0.012 | 0.644 | 0.625 | -63% | -61% | |
|  | **CC** | 0.696 | 0.041 | 0.000 | 65% | 70% | |
| **783** | **N** | 170 | 73 | 16 |  |  | |
|  | **TT** | 0.694 | 0.068 | 0.000 | 63% | 69% | |
|  | **CC** | 0.024 | 0.534 | 0.875 | -51% | -85% | |
| **7953** | **N** | 170 | 73 | 16 |  |  | |
|  | **TT** | 0.000 | 0.562 | 0.875 | -56% | -88% | |
|  | **CC** | 0.776 | 0.137 | 0.000 | 64% | 78% | |
| **797** | **N** | 170 | 73 | 16 |  |  | |
|  | **CC** | 0.041 | 0.685 | 0.938 | -64% | -90% | |
|  | **GG** | 0.665 | 0.055 | 0.000 | 61% | 66% | |
| **8220** | **N** | 167 | 73 | 16 |  |  | |
|  | **AA** | 0.599 | 0.055 | 0.000 | 54% | 60% | |
|  | **TT** | 0.006 | 0.589 | 0.750 | -58% | -74% | |
| **8288** | **N** | 171 | 72 | 16 |  |  | |
|  | **AA** | 0.596 | 0.042 | 0.000 | 55% | 60% | |
|  | **GG** | 0.012 | 0.722 | 0.938 | -71% | -93% | |
| **8883** | **N** | 170 | 73 | 16 |  |  | |
|  | **AA** | 0.712 | 0.027 | 0.000 | 68% | 71% | |
|  | **GG** | 0.029 | 0.575 | 0.875 | -55% | -85% | |
| **9097** | **N** | 171 | 73 | 16 |  |  | |
|  | **TT** | 0.018 | 0.589 | 0.563 | -57% | -54% | |
|  | **GG** | 0.684 | 0.027 | 0.000 | 66% | 68% | |
| **9302** | **N** | 171 | 73 | 16 |  |  | |
|  | **AA** | 0.094 | 0.767 | 1.000 | -67% | -91% | |
|  | **GG** | 0.480 | 0.027 | 0.000 | 45% | 48% | |
| **11344** | **N** | 171 | 73 | 16 |  |  | |
|  | **AA** | 0.813 | 0.096 | 0.313 | 72% | 50% | |
|  | **GG** | 0.012 | 0.466 | 0.125 | -45% | -11% | |
| **11541** | **N** | 171 | 73 | 16 |  |  | |
|  | **AA** | 0.000 | 0.466 | 0.125 | -47% | -13% | |
|  | **GG** | 0.784 | 0.055 | 0.250 | 73% | 53% | |
| **1177** | **N** | 170 | 73 | 16 |  |  | |
|  | **AA** | 0.024 | 0.644 | 0.063 | -62% | -4% | |
|  | **GG** | 0.582 | 0.041 | 0.625 | 54% | -4% | |
| **11805** | **N** | 167 | 73 | 16 |  |  | |
|  | **TT** | 0.814 | 0.068 | 0.313 | 75% | 50% | |
|  | **CC** | 0.006 | 0.616 | 0.438 | -61% | -43% | |
| **12151** | **N** | 169 | 72 | 16 |  |  | |
|  | **AA** | 0.556 | 0.028 | 0.188 | 53% | 37% | |
|  | **TT** | 0.006 | 0.625 | 0.500 | -62% | -49% | |
| **12915** | **N** | 171 | 73 | 16 |  |  | |
|  | **AA** | 0.573 | 0.014 | 0.438 | 56% | 14% | |
|  | **CC** | 0.035 | 0.630 | 0.125 | -60% | -9% | |
| **138** | **N** | 169 | 73 | 16 |  |  | |
|  | **AA** | 0.036 | 0.767 | 0.375 | -73% | -34% | |
|  | **GG** | 0.503 | 0.041 | 0.313 | 46% | 19% | |
| **1606** | **N** | 169 | 73 | 16 |  |  | |
|  | **CC** | 0.627 | 0.123 | 0.188 | 50% | 44% | |
|  | **GG** | 0.006 | 0.534 | 0.188 | -53% | -18% | |
| **1838** | **N** | 170 | 73 | 16 |  |  | |
|  | **AA** | 0.035 | 0.699 | 0.375 | -66% | -34% | |
|  | **GG** | 0.582 | 0.027 | 0.438 | 55% | 14% | |
| **2348** | **N** | 171 | 73 | 16 |  |  | |
|  | **TT** | 0.006 | 0.699 | 0.375 | -69% | -37% | |
|  | **CC** | 0.626 | 0.027 | 0.250 | 60% | 38% | |
| **2718** | **N** | 171 | 73 | 16 |  |  | |
|  | **AA** | 0.696 | 0.082 | 0.188 | 61% | 51% | |
|  | **GG** | 0.035 | 0.644 | 0.250 | -61% | -21% | |
| **2950** | **N** | 171 | 73 | 16 |  |  | |
|  | **TT** | 0.503 | 0.041 | 0.063 | 46% | 44% | |
|  | **CC** | 0.047 | 0.644 | 0.625 | -60% | -58% | |
| **3189** | **N** | 171 | 73 | 16 |  |  | |
|  | **AA** | 0.018 | 0.740 | 0.313 | -72% | -29% | |
|  | **CC** | 0.673 | 0.027 | 0.125 | 65% | 55% | |
| **3215** | **N** | 171 | 73 | 16 |  |  | |
|  | **TT** | 0.731 | 0.041 | 0.250 | 69% | 48% | |
|  | **CC** | 0.012 | 0.630 | 0.375 | -62% | -36% | |
| **367** | **N** | 171 | 73 | 16 |  |  | |
|  | **AA** | 0.012 | 0.740 | 0.250 | -73% | -24% | |
|  | **CC** | 0.690 | 0.041 | 0.063 | 65% | 63% | |
| **3700** | **N** | 171 | 73 | 16 |  |  | |
|  | **TT** | 0.637 | 0.041 | 0.500 | 60% | 14% | |
|  | **CC** | 0.012 | 0.699 | 0.188 | -69% | -18% | |
| **4326** | **N** | 171 | 73 | 16 |  |  | |
|  | **TT** | 0.731 | 0.041 | 0.250 | 69% | 48% | |
|  | **CC** | 0.029 | 0.616 | 0.438 | -59% | -41% | |
| **4411** | **N** | 170 | 73 | 16 |  |  | |
|  | **TT** | 0.006 | 0.575 | 0.313 | -57% | -31% | |
|  | **GG** | 0.571 | 0.055 | 0.125 | 52% | 45% | |
| **4491** | **N** | 171 | 73 | 16 |  |  | |
|  | **AA** | 0.696 | 0.068 | 0.313 | 63% | 38% | |
|  | **GG** | 0.018 | 0.562 | 0.375 | -54% | -36% | |
| **4608** | **N** | 171 | 73 | 16 |  |  | |
|  | **AA** | 0.035 | 0.562 | 0.375 | -53% | -34% | |
|  | **GG** | 0.690 | 0.082 | 0.250 | 61% | 44% | |
| **5856** | **N** | 170 | 73 | 16 |  |  | |
|  | **AA** | 0.018 | 0.644 | 0.438 | -63% | -42% | |
|  | **GG** | 0.688 | 0.096 | 0.125 | 59% | 56% | |
| **6479** | **N** | 171 | 73 | 16 |  |  | |
|  | **AA** | 0.567 | 0.041 | 0.125 | 53% | 44% | |
|  | **GG** | 0.018 | 0.616 | 0.625 | -60% | -61% | |
| **8646** | **N** | 171 | 73 | 16 |  |  | |
|  | **AA** | 0.094 | 0.575 | 0.688 | -48% | -59% | |
|  | **CC** | 0.503 | 0.041 | 0.125 | 46% | 38% | |
| **9922** | **N** | 170 | 73 | 16 |  |  | |
|  | **AA** | 0.018 | 0.616 | 0.313 | -60% | -29% | |
|  | **GG** | 0.647 | 0.055 | 0.313 | 59% | 33% | |
| **12499** | **N** | 171 | 73 | 16 |  |  | |
|  | **TT** | 0.029 | 0.288 | 0.750 | -26% | -72% | |
|  | **CC** | 0.731 | 0.233 | 0.000 | 50% | 73% | |
| **12747** | **N** | 170 | 73 | 16 |  |  | |
|  | **AA** | 0.000 | 0.315 | 0.625 | -32% | -63% | |
|  | **TT** | 0.735 | 0.205 | 0.000 | 53% | 74% | |
| **1906** | **N** | 171 | 73 | 16 |  |  | |
|  | **TT** | 0.029 | 0.205 | 0.688 | -18% | -66% | |
|  | **CC** | 0.760 | 0.315 | 0.000 | 45% | 76% | |
| **1912** | **N** | 171 | 73 | 16 |  |  | |
|  | **TT** | 0.795 | 0.411 | 0.000 | 38% | 80% | |
|  | **CC** | 0.000 | 0.096 | 0.500 | -10% | -50% | |
| **2403** | **N** | 171 | 73 | 16 |  |  | |
|  | **AA** | 0.000 | 0.301 | 0.750 | -30% | -75% | |
|  | **GG** | 0.807 | 0.178 | 0.000 | 63% | 81% | |
| **2512** | **N** | 171 | 73 | 16 |  |  | |
|  | **TT** | 0.012 | 0.233 | 0.625 | -22% | -61% | |
|  | **CC** | 0.760 | 0.425 | 0.000 | 34% | 76% | |
| **267** | **N** | 171 | 73 | 16 |  |  | |
|  | **AA** | 0.006 | 0.205 | 0.500 | -20% | -49% | |
|  | **GG** | 0.854 | 0.247 | 0.063 | 61% | 79% | |
| **3340** | **N** | 171 | 73 | 16 |  |  | |
|  | **TT** | 0.000 | 0.110 | 0.500 | -11% | -50% | |
|  | **CC** | 0.953 | 0.479 | 0.063 | 47% | 89% | |
| **3480** | **N** | 171 | 73 | 16 |  |  | |
|  | **TT** | 0.053 | 0.493 | 0.875 | -44% | -82% | |
|  | **CC** | 0.626 | 0.068 | 0.000 | 56% | 63% | |
| **3545** | **N** | 171 | 73 | 16 |  |  | |
|  | **TT** | 0.053 | 0.438 | 0.688 | -39% | -63% | |
|  | **CC** | 0.474 | 0.123 | 0.000 | 35% | 47% | |
| **3656** | **N** | 171 | 73 | 16 |  |  | |
|  | **AA** | 0.000 | 0.205 | 0.875 | -21% | -88% | |
|  | **GG** | 0.854 | 0.260 | 0.000 | 59% | 85% | |
| **3952** | **N** | 171 | 73 | 16 |  |  | |
|  | **AA** | 0.018 | 0.151 | 0.688 | -13% | -67% | |
|  | **GG** | 0.632 | 0.370 | 0.000 | 26% | 63% | |
| **4001** | **N** | 171 | 73 | 16 |  |  | |
|  | **TT** | 0.012 | 0.411 | 0.750 | -40% | -74% | |
|  | **CC** | 0.737 | 0.082 | 0.063 | 65% | 67% | |
| **4108** | **N** | 171 | 73 | 16 |  |  | |
|  | **AA** | 0.591 | 0.534 | 0.063 | 6% | 53% | |
|  | **GG** | 0.035 | 0.096 | 0.500 | -6% | -46% | |
| **4294** | **N** | 171 | 73 | 16 |  |  | |
|  | **AA** | 0.749 | 0.233 | 0.125 | 52% | 62% | |
|  | **GG** | 0.012 | 0.329 | 0.500 | -32% | -49% | |
| **5183** | **N** | 171 | 73 | 16 |  |  | |
|  | **TT** | 0.462 | 0.041 | 0.000 | 42% | 46% | |
|  | **CC** | 0.146 | 0.644 | 0.875 | -50% | -73% | |
| **539** | **N** | 171 | 73 | 16 |  |  | |
|  | **AA** | 0.006 | 0.260 | 0.875 | -25% | -87% | |
|  | **GG** | 0.924 | 0.260 | 0.000 | 66% | 92% | |
| **5449** | **N** | 171 | 73 | 16 |  |  | |
|  | **TT** | 0.035 | 0.397 | 0.875 | -36% | -84% | |
|  | **CC** | 0.649 | 0.110 | 0.000 | 54% | 65% | |
| **5601** | **N** | 170 | 73 | 16 |  |  | |
|  | **TT** | 0.018 | 0.397 | 0.813 | -38% | -79% | |
|  | **CC** | 0.747 | 0.123 | 0.000 | 62% | 75% | |
| **7131** | **N** | 171 | 73 | 16 |  |  | |
|  | **AA** | 0.047 | 0.342 | 0.688 | -30% | -64% | |
|  | **GG** | 0.462 | 0.205 | 0.000 | 26% | 46% | |
| **749** | **N** | 171 | 73 | 16 |  |  | |
|  | **AA** | 0.058 | 0.027 | 0.750 | 3% | -69% | |
|  | **TT** | 0.544 | 0.671 | 0.000 | -13% | 54% | |
| **751** | **N** | 171 | 73 | 16 |  |  | |
|  | **AA** | 0.848 | 0.219 | 0.000 | 63% | 85% | |
|  | **GG** | 0.006 | 0.356 | 0.750 | -35% | -74% | |
| **754** | **N** | 170 | 73 | 16 |  |  | |
|  | **AA** | 0.118 | 0.753 | 1.000 | -64% | -88% | |
|  | **CC** | 0.459 | 0.027 | 0.000 | 43% | 46% | |
| **761** | **N** | 171 | 73 | 16 |  |  | |
|  | **AA** | 0.047 | 0.658 | 0.938 | -61% | -89% | |
|  | **GG** | 0.503 | 0.068 | 0.000 | 43% | 50% | |
| **8198** | **N** | 170 | 73 | 16 |  |  | |
|  | **AA** | 0.753 | 0.315 | 0.000 | 44% | 75% | |
|  | **CC** | 0.024 | 0.205 | 0.875 | -18% | -85% | |
| **9524** | **N** | 170 | 73 | 16 |  |  | |
|  | **AA** | 0.676 | 0.192 | 0.063 | 48% | 61% | |
|  | **TT** | 0.006 | 0.329 | 0.563 | -32% | -56% | |
| **9722** | **N** | 171 | 73 | 16 |  |  | |
|  | **TT** | 0.006 | 0.342 | 0.500 | -34% | -49% | |
|  | **CC** | 0.813 | 0.123 | 0.125 | 69% | 69% | |
| **1372** | **N** | 171 | 73 | 16 |  |  | |
|  | **TT** | 0.000 | 0.082 | 0.750 | -8% | -75% | |
|  | **CC** | 0.906 | 0.411 | 0.000 | 50% | 91% | |
