# Supplementary material for: EST–SNP Study of Olea europaea L. Uncovers Functional Polymorphisms between Cultivated and Wild Olives
Source: Genes (Basel). 2020 Aug 10;11(8):916. doi: 10.3390/genes11080916 (PMC7465833; doi:10.3390/genes11080916)
Supplement: Supplementary file 1 [file genes-11-00916-s001.zip › Table_S5.docx]

**Table S5.** List of the 124 genes differentiating olive cultivars from wild olives and subsp. *guanchica* samples. Name of contigs, linkage group placement, NCBI accession number, gene name and molecular process and function for each gene are reported.

| **Contig EST-SNP** | **Chromosomes*** | **NCBI Accession/ Gene Number** | **Synonymous/ Non-synonyous** | **Gene Name** | **Cellular Component** | **Biological Process & Function** | **Molecular Function** |
| --- | --- | --- | --- | --- | --- | --- | --- |
| **10026** | **7** | LOC111397552 | syn | **Galactan beta-1,4-galactosyltransferase, GALS1** | Golgi apparatus | Pectin biosynthetic process, macromolecule biosynthetic process, cell wall biogenesis, cellular component biogenesis | Beta-N-acetylglucosaminylglycopeptide beta-1,4-galactosyltransferase activity-transferase activity, transferring glycosyl groups |
| **10109** | **1** | LOC111399950 | syn | **Elongin-C-like** | Cytoplasm and nucleus | Important host factor involved in SCMV accumulation, ZmElc may be hijacked by the virus from its normal role in the plant to act as an enhancer during viral RNA replication. Primer for viral RNA replication, and the accumulation of SCMV RNA is increased when ZmELC is transiently overexpressed in maize protoplasts | *Not defined* |
| **10266** | **11** | LOC111403214 | syn | **TIFY 6B or JAZ3 jasmonate-zim-domain protein 3** | Nucleus | Defense response, jasmonic acid mediated signaling pathway, regulation of defense response, regulation of jasmonic acid mediated signaling pathway, response to wounding | Protein binding |
| **10405** | **18** | LOC111367357 | syn | **Probable fructokinase 6** | Cytoplasm, cytosol, plasmodesma | Fructose metabolic process, starch biosynthetic process | ATP binding, carbohydrate kinase activity, fructokinase activity, kinase activity, zinc ion binding |
| **10411** | **14** | LOC111385514 | 3' UTR | **V-type proton ATPase 16** | Chloroplast, integral component of membrane, plant-type vacuole membrane, vacuolar membrane, vacuole | Calcium ion transmembrane transport, calcium ion transport, cellular calcium ion homeostasis, cellular manganese ion homeostasis, cellular zinc ion homeostasis, cold acclimation, phosphate ion homeostasis, response to salt stress | Calcium ion transmembrane transporter activity, calcium: cation antiporter activity, calcium: proton antiporter activity, protein binding |
| **10481** | **22** | LOC111371775 | from Y to A | **Reticulon-like protein B2** | Golgi apparatus, endoplasmic reticulum, nucleus, plasma membrane | Endoplasmic reticulum tubular network organization | *not defined* |
| **1064** | **18** | LOC111387859 | syn | **Iron-sulfur assembly protein IscA, chloroplastic-like, SUFA** | Chloroplast, chloroplast stroma | Iron-sulfur cluster assembly, protein maturation by iron-sulfur cluster transfer | Iron-sulfur cluster binding, metal ion binding, structural molecule activity |
| **10672** | **20** | LOC111369664 | syn | **Serine/arginine repetitive matrix protein 1** | Nucleus, spliceosomal complex | Biological process GO:0008150 | Molecular function GO:0003674 |
| **11058** | **16** | LOC111392792 | 3' UTR | **Heterogeneous nuclear ribonucleoprotein Q-like** | Cytoplasm, cytosol, endoplasmic reticulum, nucleus | Cellular response to jasmonic acid stimulus, defense response to bacteria and fungi, innate immune response, multicellular organism development, negative regulation of innate immune response, positive regulation of transcription, DNA-templated, regulation of cell fate determination, flower development, gene expression, glucosinolate biosynthetic process, histone H3-K27 trimethylation, response to bacteria | Chromatin binding, protein binding, sequence-specific DNA binding |
| **11296** | **2** | LOC111392247 | syn | **U-box domain-containing protein 15-like** | Cytoplasm | Protein ubiquitination | Ubiquitin-protein transferase activity |
| **11541_2** | **17** | PON65285.1 | from G to A | **Neuronal acetylcholine receptor subunit alpha, predicted** | Integral component of membrane, mitochondrial membrane, mitochondrial respiratory chain complex I, mitochondrion, respiratory chain complex I | Photorespiration | Molecular function GO:0003674 |
| **11650** | **16** | LOC111412388 | syn | **Photolyase/blue-light receptor 2, PHR2** | Nucleus | DNA repair, protein-chromophore linkage, response to stimulus | DNA photolyase activity, photoreceptor activity |
| **11682** | **1** | LOC111402594 | syn | **60S Ribosomal protein L23-like** | Cell wall, cytoplasm, cytosol, cytosolic large ribosomal subunit, cytosolic ribosome, intracellular, nucleolus, plasmodesma | Response to cold, high light intensity and oxidative stress, ribosomal large subunit assembly, ribosome biogenesis, translation | rRNA binding, structural constituent of ribosome |
| **11788** | **1** | LOC111381964 | syn | **Activating signal cointegrator 1, CPK1** | Cytoplasm, membrane, nucleus, peroxisomal membrane, peroxisome, plasma membrane | Abscisic acid-activated signaling pathway, intracellular signal transduction, peptidyl-serine phosphorylation, protein autophosphorylation, protein phosphorylation | ATP binding, calcium ion binding, calcium-dependent protein kinase activity, calcium-dependent protein serine/threonine kinase activity, calmodulin binding, calmodulin-dependent protein kinase activity, kinase activity, protein serine/threonine kinase activity |
| **12110** | **Unplaced** | XM_009620635.2 | syn | **Putative transmembrane protein** | Transmembrane protein | *Not defined* | *Not defined* |
| **135** | **Unplaced** | LOC111385303 | 5' UTR | **Protein ECERIFERUM 3-like** | Endoplasmic reticulum membrane, integral component of membrane, membrane, plasma membrane | Alkane biosynthetic process, cuticle development, cuticle hydrocarbon biosynthetic process, oxidation-reduction process, pollen sperm cell differentiation, sphingolipid biosynthetic process, wax biosynthetic process | Aldehyde oxygenase (deformylating) activity, catalytic activity, iron ion binding, octadecanal decarbonylase activity, sphingosine hydroxylase activity |
| **1395** | **10** | LOC111401945 | syn | **Peroxidase 72-like** | Extracellular region | Hydrogen peroxide catabolic process, lignin biosynthetic process, oxidation-reduction process, response to oxidative stress | Heme binding, metal ion binding, peroxidase activity |
| **1650** | **Unplaced** | LOC111382810 | syn | **14-3-3 family protein** | Chloroplast | Involved in flowering stage, petal differentiation and expansion stage | Protein phosphorylated amino acid binding |
| **1684** | **16** | LOC111411888 | syn | **DAD1-like acylhydrolase, Phospholipase A1 gamma3, DLAH** | Extracellular region, mitochondrion | Lipid metabolic process, seed coat development | Phospholipase A1 activity, phospholipase activity, triglyceride lipase activity |
| **1775** | **22** | LOC111371815 | syn | **General transcription factor IIE subunit 2-like** | Nucleus | Regulation of transcription, DNA-templated | DNA binding |
| **2011** | **Unplaced** | LOC111386790 | from G to S | **Probable pectinesterase/pectinesterase inhibitor, PMEI34** | Membrane | Cell wall biogenesis-degradation, stress response to fungus (*Verticillium*) | Aspartyl esterase, Hydrolase |
| **2040** | **21** | LOC111370402 | from H to Q | **Xyloglucan endotransglucosylase/hydrolase protein 9-like, XTH9** | Apoplast, cell wall, extracellular region | Cell wall biogenesis, cell wall organization, xyloglucan metabolic process | Hydrolase activity, acting on glycosyl bonds, hydrolase activity, Hydrolyzing O-glycosyl compounds, xyloglucan: xyloglucosyl transferase activity |
| **208** | **12** | LOC111369915 | syn | **Cell division cycle protein 48 homolog, CDC48** | Golgi apparatus, apoplast, cell wall, cytoplasm, cytosol, cytosolic ribosome, lipid droplet, nucleolus, nucleus, membrane, plasmodesma, spindle | Phosphorylation | ATP binding, ATPase activity, identical protein binding, polyubiquitin modification-dependent protein binding, protein binding |
| **2104** | **Unplaced** | LOC111388219 | 3'UTR | **Glycerol-3-phosphate sn-2-acyltransferase 4, GPAT4** | Chloroplast, integral component of membrane, membrane | Cutin biosynthetic process, phospholipid biosynthetic process | Glycerol-3-phosphate 2-O-acyltransferase activity, phosphatase activity, sn-2-glycerol-3-phosphate omega-OH-C22:0-CoA acyl transferase activity, transferase activity, transferring acyl groups |
| **2159** | **15** | KU701103.1 | 3'UTR | **Adagio 2 or lov kelch protein 2, Putative light–oxygen–voltage sensitive domain containing protein** | Cajal body, SCF ubiquitin ligase complex, cytosol, nucleus | Circadian rhythm, flower development, protein ubiquitination, protein-chromophore linkage, regulation of circadian rhythm, response to blue light, ubiquitin-dependent protein catabolic process | Photoreceptor activity, protein binding, ubiquitin-protein transferase activity |
| **2331** | **Unplaced** | LOC111377989 | syn | **Magnesium-chelatase subunit, ChlH** | Chloroplast | Photosynthesis, chlorophyll biosynthetic process | ATP binding, magnesium chelatase activity |
| **2607** | **11** | LOC111403023 | 3'UTR | **Vacuolar-sorting receptor 1-like** | Membrane | Calcium ion binding, receptor | calcium ion binding, receptor |
| **2690** | **Unplaced** | LOC111386875 | 3'UTR | **Geraniol dehydrogenase 1-like, GEDH1** | *Not defined* | Oxidation-reduction process, involved in the production of citral, a mixture of geranial and neral with a strong lemony scent. Reversibly oxidizes geraniol and nerol in equal efficiency | Geraniol dehydrogenase activity, zinc ion binding, Oxidoreductase |
| **2724** | **Unplaced** | LOC111378038 | syn | **Cysteine protease, RD19A-like** | Extracellular region, extracellular space, lysosome, lytic vacuole, nucleus, vacuole | Defense response to bacterium, proteolysis, proteolysis involved in cellular protein catabolic process, response to desiccation, osmotic stress, salt stress and water deprivation | Cysteine-type endopeptidase activity, cysteine-type peptidase activity |
| **2748** | **17** | LOC111366133 | 5'UTR | **Profilin-2-lik, PRF2** | Actin cytoskeleton, cell cortex, chloroplast, cytoplasm, cytoskeleton, plasma membrane | Actin polymerization or depolymerization, cytoskeleton organization, inflorescence development, lateral root development, leaf development, sequestering of actin monomers | Actin monomer binding, protein binding |
| **2824** | **20** | LOC111369836 | syn | **Stress-related protein-like, SRP** | Embryo | Post germinative growth, diurnal regulation of lipid droplets and various stress responses | Phosphoprotein |
| **288** | **1** | LOC111398431 | syn | **Acetyl co-enzyme a carboxylase biotin carboxylase subunit, CAC2** | Chloroplast, chloroplast envelope, chloroplast stroma | Fatty acid biosynthetic process, malonyl-CoA biosynthetic process | ATP binding, acetyl-CoA carboxylase activity, biotin carboxylase activity, metal ion binding |
| **3022** | **2** | LOC111371758 | from A to V | **UDP-glucose 6-dehydrogenase 3-like, UGD3** | Cell wall, cytoplasm, cytosol, nucleus | UDP-glucuronate biosynthetic process, carbohydrate metabolic process, cell wall pectin metabolic process, glycosaminoglycan biosynthetic process, oxidation-reduction process | NAD binding, UDP-glucose 6-dehydrogenase activity |
| **3158** | **11** | LOC111404241 | syn | **60S ribosomal protein L9-like, RPL9B** | Chloroplast, chloroplast envelope, chloroplast stroma, mitochondrion, ribosome | Ribosome biogenesis, translation | Structural constituent of ribosome, rRNA binding |
| **319** | **Unplaced** | LOC111389816 | syn | **Geraniol synthase chloroplastic-like, GES** | Chloroplast | Geranyl diphosphate metabolic process, pathway of terpenoid biosynthesis | Hydrolase activity, terpene synthase activity |
| **3236** | **17** | LOC111365482 | from R to Q | **Fatty acid amide hydrolase-like** | Golgi apparatus, endoplasmic reticulum, endoplasmic reticulum membrane, integral component of membrane, plasma membrane, vacuolar membrane, vacuole | N-acylethanolamine metabolic process, defense response to bacterium | N-(long-chain-acyl)ethanolamine deacylase activity, amidase activity, anandamide amidohydrolase activity, oleamide hydrolase activity |
| **344** | **Unplaced** | LOC111391315 | from G to S | **60S acidic ribosomal protein, P2B-like** | Cytosol, cytosolic ribosome, nucleus, ribosome | Translational elongation | Structural constituent of ribosome |
| **3481** | **12** | LOC111405311 | syn | **Pectate lyase A10** | Membrane | Pectin catabolic process, response to nematode | Metal ion binding, pectate lyase activity |
| **3549** | **6** | LOC111395330 | 3'UTR | **Peptide methionine sulfoxide reductase-like** | Nucleus | Biological process GO:0008150 | Molecular function GO:0003674 |
| **3597** | **12** | LOC111405677 | 5'UTR | **60S ribosomal protein L13-1** | Cytoplasm | Response to cytokinin, translation | mRNA binding, structural constituent of ribosome |
| **3601** | **14** | LOC11140887 | 3'UTR | **Ethylene-responsive transcription factor, RAP2-4-like** | Nucleus | Cellular response to abiotic stress, probably acts as a transcriptional activator, binds to the GCC-box pathogenesis-related promoter element, may be involved in the regulation of gene expression by stress factors and by components of stress signal transduction pathways | Sequence-specific DNA binding |
| **3710** | **10** | LOC111400936 | syn | **Agamous-like MADS-box protein, AGL8 homolog** | Nucleus | Positive regulation of flower development, probable transcription factor that promotes early floral meristem identity in synergy with APETALA1 and CAULIFLOWER, or partially redundant to their function in the up-regulation of LEAFY. It is required subsequently for the transition of an inflorescence meristem into a floral meristem, it is also required for normal pattern of cell division, expansion and differentiation during morphogenesis of the silique, represses SAUR10 expression in stems and inflorescence branches | RNA polymerase II regulatory region sequence-specific DNA binding |
| **4188** | **20** | LOC111369072 | 3'UTR | **Outer envelope pore protein 16 chloroplastic, OEP16-1** | Chloroplast | Ion transport, transport, voltage-dependent high-conductance channel with a slight cation-selectivity, selective for amino acids but excludes triosephosphates or uncharged sugars, Non-essential amino acid-selective channel protein and translocation pore for NADPH: protochlorophyllide oxidoreductase A (PORA) and possibly PORB, involved in PORA precursor (pPORA) import and thus confers photoprotection onto etiolated seedlings during greening | Porin |
| **4374** | **Unplaced** | LOC111387830 | from E to G | **Dehydrin, HIRD11** | Nucleus | Negative regulation of hydrogen peroxide biosynthetic process, intrinsically disordered and metal-binding protein. Binds to the divalent cations cobalt, nickel, copper and zinc, but not to magnesium, calcium, manganese or cadmium, binding to metal ions decreases disordered state, decreases susceptibility to trypsin and promotes self-association, can reduce the formation of reactive oxygen species (ROS) in a copper-ascorbate in vitro system | Cobalt, copper, nickel, zinc ion binding |
| **4375** | **Unplaced** | XM_020254215.1 | syn | **Galactan beta-1,4-galactosyltransferase, GALS1-like** | Golgi apparatus | Cell wall biogenesis/degradation, involved in the biosynthesis of beta-1,4-galactan, can transfer galactose residues from UDP-galactose to beta-1,4-galactopentaose in vitro, forms specifically beta-1,4-galactosyl linkages and can add successive beta-1,4-galactosyl residues to the acceptor. Beta-1,4-galactans are abundant polysaccharides in plant cell walls and are found as side-chain of rhamnogalacturonan I, which is a major component of pectin | Beta-1,3-galactosyltransferase activity |
| **4534** | **Unplaced** | LOC111379443 | syn | **Chalcone synthase J-like, CHS** | Endoplasmic reticulum, Nucleus, Vacuole | Flavonoid biosynthesis, the primary product of this enzyme is 4,2',4',6'-tetrahydroxychalcone (also termed naringenin-chalcone or chalcone) which can under specific conditions spontaneously isomerize into naringenin | Naringenin-chalcone synthase activity |
| **4599** | **12** | LOC111405278 | 3'UTR | **Gibberellin-regulated protein 14-like, GASA14** | Extracellular region or secreted | Gibberellin signaling pathway, response to salt stress, gibberellin-regulated protein that may function in hormonal controlled steps of development such as seed germination, flowering and seed maturation | Gibberellic acid mediated signaling pathway |
| **4714** | **Unplaced** | LOC111385836 | syn | **Polyubiquitin-like** | Nucleus | Ubiquitin-dependent protein catabolic process, ubiquitin is encoded by 16 different genes, ubiquitin is generally synthesized as a polyubiquitin precursor with tandem head to tail repeats, often, there is one to three additional amino acids after the last repeat, removed in the mature protein | Ubiquitin protein ligase binding |
| **4794** | **Unplaced** | LOC111392260 | from H to Q | **Transmembrane protein 64-like** | Integral component of membrane | *Not defined* | *Not defined* |
| **4851** | **3** | LOC111390476 | 5'UTR | **Protein Curvature Thylakoid 1A, chloroplastic-like** | Chloroplast | Coiled coil, transit peptide, transmembrane, transmembrane helix | Coiled coil, Transit peptide, Transmembrane, Transmembrane helix |
| **504** | **Unplaced** | LOC111379232 | syn | **Geraniol 8-hydroxylase-like** | Endoplasmic reticulum | Monoterpenoid biosynthetic process, hydroxylase involved in the biosynthesis of hydroxygeraniol, a precursor of the terpenoid indole alkaloids such as vinblastine and vincristine, also able to hydroxylate in vitro nerol and to catalyze 3'-hydroxylation of the flavanone naringenin to form eriodictyol. No activity with apigenin, kaempferol, p-coumaric acid and ferulic acid as substrates | Heme binding, oxidoreductase activity, acting on paired donors, with incorporation or reduction of molecular oxygen, NAD(P)H as one donor, and incorporation of one atom of oxygen |
| **5193** | **8** | LOC111398497 | syn | **Ubiquitin-conjugating enzyme, E2-4-like** | Nucleus | Endosperm development, accepts the ubiquitin from the E1 complex and catalyzes its covalent attachment to other proteins. Mediates the selective degradation of short-lived and abnormal proteins | ATP binding, ubiquitin conjugating enzyme activity |
| **5398** | **Unplaced** | LOC111381097 | syn | **40S ribosomal protein S23** | Cytosolic small ribosomal subunit, Nucleus, polysomal ribosome | Translation | Structural constituent of ribosome |
| **5822** | **10** | LOC111401120 | syn | **Photosystem I reaction center subunit N chloroplastic-like, PSAN** | Chloroplast thylakoid membrane | Photosynthesis, may function in mediating the binding of the antenna complexes to the PSI reaction center and core antenna, plays an important role in docking plastocyanin to the PSI complex, does not bind pigments | Protein domain specific binding |
| **6061** | **21** | PSS06480.1 | syn | **Glycerol-3-phosphate acyltransferase, GPAT** | Endoplasmic reticulum membrane | Fatty acid biosynthetic process, gametophyte development, essential protein required for male and female gametophytes development, exhibits sn-1 acyltransferase activity with high specificity for acyl-coenzyme A, thus triggering storage lipid biosynthesis and playing an important role in the Kennedy pathway of glycerolipid biosynthesis, catalyzes triacylglycerol (TAG) accumulation involved in membrane lipid and oil biosynthesis, especially in seeds, contributes also to the biosynthesis of both polar lipids and TAG in developing leaves, as well as lipid droplet production in developing pollen grains, seems to not contribute to surface lipid biosynthesis (e.g. waxes and cutin) | Glycerol-3-phosphate O-acyltransferase activity, protein self-association |
| **6453** | **Unplaced** | LOC111382118 | 3'UTR | **FT-interacting protein 1-like** | Endoplasmic reticulum membrane, plasmodesma | Long-day photoperiodism, flowering, cellular protein localization, involved in the export of FT from the phloem companion cells to the sieve elements through the plasmodesmata, regulates flowering time under long days | Protein transporter activity |
| **6604** | **10** | LOC111402460 | syn | **Ubiquitin thioesterase, OTU1-like** | Nucleus | Protein K48-linked deubiquitination | Ubiquitin binding, thiol-dependent ubiquitin-specific protease activity, NEDD8-specific protease activity |
| **662** | **22** | LOC111371391 | syn | **Tubulin alpha-3 chain, TUBA3** | Cytoskeleton | Tubulin is the major constituent of microtubules, it binds two moles of GTP, one at an exchangeable site on the beta chain and one at a non-exchangeable site on the alpha chain | GTPase activity, GTP binding, structural constituent of cytoskeleton |
| **686** | **Unplaced** | LOC111379114 | syn | **Glucose-6-phosphate/phosphate translocator 2 chloroplastic-like, GPT2** | Chloroplast | Glucose-6-phosphate transport, glucose 6-phosphate (Glc6P) transporter, transports also inorganic phosphate, 3-phosphoglycerate, triose phosphates and, to a leser extent, phosphoenolpyruvate, responsible for the transport of Glc6P into plastids of heterotrophic tissues where it can be used as a carbon source for starch biosynthesis, as substrate for fatty acid biosynthesis or as substrate for NADPH generation via the oxidative pentose phosphate pathway (OPPP), required for dynamic acclimation of photosynthesis | Inorganic phosphate transmembrane transporter activity |
| **6913** | **4** | LOC111393211 | from K to R | **Serine/threonine-protein kinase, WNK4** | Nucleus, cytoplasm | Intracellular signal transduction, protein autophosphorylation, was named WNK with no lysine(K) because key residues for catalysis, including the lysine involved in ATP binding, are either not conserved or differ compared to the residues described in other kinase family proteins | ATP binding, protein serine/threonine kinase activity |
| **6929** | **6** | LOC111395671 | from R to K | **Protein translation factor, SUI1 homolog** | *Not defined* | Regulation of translation, increase floral organs | Translation initiation factor activity |
| **722** | **23** | LOC111372393 | from L to Q | **Serine carboxypeptidase-like 7, SCPL** | Extracellular region or secreted | Secondary metabolic process | Serine-type carboxypeptidase activity |
| **7348** | **18** | LOC111367905 | syn | **S-adenosylmethionine decarboxylase proenzyme-like, SAMDC** | Cytosol | Gene silencing, plant organ development, Spermidine biosynthesis, essential for biosynthesis of the polyamines spermidine and spermine. Essential for polyamine homeostasis, and normal plant embryogenesis, growth and development | Adenosylmethionine decarboxylase activity |
| **7809** | **Unplaced** | LOC111375008 | from D to T | **Protein networked 1A-like, NET1A** | Cytoskeleton, Cell membrane, plasmodesma | Plant-specific actin binding protein, associates with F-actin at the plasma membrane and plasmodesmata, may be part of a membrane-cytoskeletal adapter complex | Actin filament binding |
| **783** | **11** | LOC111404219 | syn | **Tripeptidyl-peptidase 2-like** | Chloroplast, cytosolic ribosome, vacuolar membrane, cytoplasm, membrane | Proteolysis, serine protease of the proteasome pathway that may function with the 20S proteasome to degrade oxidized proteins generated by environmental stress | Serine-type endopeptidase activity |
| **7953** | **15** | LOC111410264 | 5'UTR | **Luc7-like protein 3** | Nucleus | mRNA splice site selection | mRNA binding |
| **797** | **Unplaced** | LOC111377592 | syn | **Tetraketide alpha-pyrone reductase 1-like, TKPR1** | Nucleus, endoplasmic reticulum, cytoplasm | Pollen development, sporopollenin biosynthetic process, involved in the biosynthesis of hydroxylated tetraketide compounds that serve as sporopollenin precursors (the main constituents of exine), essential for pollen wall development, acting on tetraketide alpha-pyrones and reducing the carbonyl function on the tetraketide alkyl chain to a secondary alcohol function | Coenzyme binding, oxidoreductase activity |
| **8220** | **18** | OVA16924.1 | from E to V | **DSBA-like thioredoxin domain, BVC80_9049g60** | *Not defined* | *Not defined* | Protein disulfide oxidoreductase activity |
| **8288** | **4** | PSS01414.1 | 3'UTR | **Protein RNA-directed DNA methylation like, RdDM** | Nucleus, nucleoplasm | RNA-mediated gene silencing, production of siRNA involved in RNA interference, effector of RNA-directed DNA methylation (RdDM) triggered by small interfering RNAs (siRNAs, 24-nt RNAs), functions as an adapter protein that binds scaffold transcripts generated by polymerase V and recruits AGO4 and AGO4-bound siRNAs to form an RdDM effector complex, promotes the expression of 24-nt RNAs, required for the initial establishment of DNA methylation, together with AGO4, required for transcriptional gene silencing (TGS) by DNA methylation and repressive histone modifications (H3K9me2) of several chromatin loci | Binding |
| **8883** | **1** | LOC111396805 | from M to V | **Heat shock 70 kDa protein 14-like, HSP70-14** | Nucleus, cytoplasm | Response to heat, response to cadmium ion, in cooperation with other chaperones, Hsp70s stabilize preexistent proteins against aggregation and mediate the folding of newly translated polypeptides in the cytosol as well as within organelles, these chaperones participate in all these processes through their ability to recognize nonnative conformations of other proteins, they bind extended peptide segments with a net hydrophobic character exposed by polypeptides during translation and membrane translocation, or following stress-induced damage | ATP binding |
| **9097** | **Unplaced** | XM_026568485.1 | syn | **RGS1-HXK1-interacting protein 1-like** | Mitochondrion | Required for some glucose-regulated gene expression, being a physical connection between RGS1 and HXK1 in sugar signaling, prevents roots and inflorescences growth | Regulation of glucose mediated signaling pathway |
| **9302** | **Unplaced** | LOC111378736 | from S to T | **Serine/threonine-protein kinase, WNK9** | Nucleus, cytoplasm | Intracellular signal transduction, protein autophosphorylation, was named WNK with no lysine(K) because key residues for catalysis, including the lysine involved in ATP binding, are either not conserved or differ compared to the residues described in other kinase family proteins | ATP binding, protein serine/threonine kinase activity |
| **11344** | **12** | LOC111405363 | 3'UTR | **Biotin carboxyl carrier protein of acetyl-CoA carboxylase 1 chloroplastic-like, BCCP1** | Chloroplast | Fatty acid biosynthetic process, this protein is involved in the pathway of fatty acid biosynthesis, which is part of lipid metabolism, this protein is a component of the acetyl coenzyme A carboxylase complex; first, biotin carboxylase catalyzes the carboxylation of the carrier protein and then the transcarboxylase transfers the carboxyl group to form malonyl-CoA | Acetyl-CoA carboxylase activity |
| **11541_1** | **17** | PON72567.1 | 3'UTR | **Neuronal acetylcholine receptor subunit alpha, At2g31490** | Mitochondrial respiratory chain complex I, integral component of membrane | Photorespiration | Receptor |
| **1177** | **Unplaced** | LOC111378472 | syn | **Hypersensitive-induced response protein-like protein, HIR** | Cell membrane | Positive regulator of hypersensitive response (HR)-like cell death, may be involved in potassium ion channel regulation | Protein histidine kinase binding |
| **11805** | **Unplaced** | PWA73961.1 | 3'UTR | **Phospholipase-like protein, At4g38560** | Nucleus | Double-strand break repair, replication-born double-strand break repair via sister chromatid exchange | Chromatin binding |
| **12151** | **1** | LOC111400494 | 3'UTR | **ABC transporter F family member 1, ABCF1** | Plasma membrane | Transport | ATPase activity |
| **12915** | **3** | LOC111386617 | 3'UTR | **30S ribosomal protein 2, chloroplastic** | Chloroplast | Stress response, component of the chloroplast ribosome (chloro-ribosome), a dedicated translation machinery responsible for the synthesis of chloroplast genome-encoded proteins, including proteins of the transcription and translation machinery and components of the photosynthetic apparatus, may have a role in the recruitment of stored chloroplast mRNAs for active protein synthesis, regulates negatively resistance responses to abiotic stresses during seed germination (e.g. salt, dehydration, and low temperature) and seedling growth (e.g. salt) | Chaperone, DNA-binding, Ribonucleoprotein, Ribosomal protein, RNA-binding, rRNA-binding |
| **138** | **7** | LOC11139736 | syn | **Elongation factor 1-beta-like** | Plasma membrane | Protein biosynthesis | TRanslation elongation factor activity |
| **1606** | **16** | LOC111411923 | syn | **Probable ubiquitin-like-specific protease 2A, ULP2A** | *Not defined* | Protein desumoylation, SUMO pathway modifies hundreds of proteins that participate in diverse cellular processes, SUMO pathway is the most studied ubiquitin-like pathway that regulates a wide range of cellular events, evidenced by a large number of sumoylated proteins identified in more than ten large-scale studies | SUMO-specific endopeptidase activity |
| **1838** | **Unplaced** | LOC111375915 | syn | **Bidirectional sugar transporter N3-like** | Multi-pass membrane protein | Sugar transport, transport, mediates both low-affinity uptake and efflux of sugar across the plasma membrane | Sugar transmembrane transporter activity |
| **2348** | **4** | LOC111393458 | from S to L | **Transcription factor 56-like, NAC** | Nucleus | Integument development, seed morphogenesis, transcription factor of the NAC family, together with NAC018/NARS2, regulates embryogenesis by regulating the development and degeneration of ovule integuments, a process required for intertissue communication between the embryo and the maternal integument | Transcription regulatory region DNA binding |
| **2718** | **Unplaced** | LOC111381423 | syn | **4-hydroxy-3-methylbut-2-enyl diphosphate reductase, chloroplastic-like, ISPH** | Chloroplast stroma | Isopentenyl diphosphate biosynthetic process, methylerythritol 4-phosphate pathway, enzyme of the plastid non-mevalonate pathway for isoprenoid biosynthesis that converts 1-hydroxy-2-methyl-2-(E)-butenyl 4-diphosphate into isopentenyl diphosphate (IPP) and dimethylallyl diphosphate (DMAPP), is essential for chloroplast development. | Oxidoreductase, metal ion binding |
| **2950** | **Unplaced** | KJ418410 | syn | **Geraniol 10-hydroxylase, G10H** | Endoplasmic reticulum | Monoterpenoid biosynthetic process, hydroxylase involved in the biosynthesis of hydroxygeraniol, a precursor of the terpenoid indole alkaloids such as vinblastine and vincristine, also able to hydroxylate in vitro nerol and to catalyze 3'-hydroxylation of the flavanone naringenin to form eriodictyol, no activity with apigenin, kaempferol, p-coumaric acid and ferulic acid as substrates | Heme binding, oxidoreductase activity, acting on paired donors, with incorporation or reduction of molecular oxygen |
| **3189** | **22** | LOC111371488 | syn | **Aquaporin, TIP2-1-like** | Vacuole membrane | Water transport, aquaporin required to facilitate the transport of water from the vacuolar compartment to the cytoplasm | Identical protein binding, methylammonium transmembrane transporter activity |
| **3215** | **10** | LOC111401925 | syn | **Probable serine incorporator** | Transmembrane helix | Transmembrane helix | Transmembrane helix |
| **367** | **22** | LOC111371766 | syn | **Catalase isozyme 3-like, CAT3** | Mitochondrion | Circadian rhythm, cold acclimation, response to oxidative stress, occurs in almost all aerobically respiring organisms and serves to protect cells from the toxic effects of hydrogen peroxide, its levels are highest in the light period and are lowest in the dark period, hence it may be important for scavenging hydrogen peroxide at night, rather than during the day. | Catalase activity |
| **3700** | **23** | LOC111372154 | syn | **Esterase/lipase, GDSL At2g04570-like** | Extracellular region or secreted | lipid catabolic process | Hydrolase activity, acting on ester bonds |
| **4326** | **Unplaced** | LOC111386437 | from N to D | **Eukaryotic peptide chain release factor subunit 1-3-like, ERF1-3** | Cytoplasm | Cytoplasmic translational termination, directs the termination of nascent peptide synthesis (translation) in response to the termination codons UAA, UAG and UGA, modulates plant growth and development | Translation release factor activity, codon specific |
| **4411** | **1** | LOC111377387 | from D to E | **Two-component response regulator-like, PRR37** | Nucleus | Biological rhythms, flowering, phytochrome signaling pathway, transcription regulation, two-component regulatory system, probable transcription factor involved in the regulation of flowering time under long day (LD) conditions, functions as repressor of flowering, controls flowering time by negatively regulating the expression of HD3A, acts downstream of the phytochrome B to repress the expression of EHD1, an activator of the flowering promoter genes HD3A and RFT1, controls photoperiodic flowering response, seems to be one of the component of the circadian clock, expression of several members of the ARR-like family is controlled by circadian rhythm, the particular coordinated sequential expression of PRR73, PRR37, PRR95, PRR59 and PPR1 result to circadian waves that may be at the basis of the endogenous circadian clock | Transcription |
| **4491** | **Unplaced** | FJ882981.1 | syn | **SnRK2 calcium sensor, SCaS** | *Not defined* | *Not defined* | Calcium ion binding |
| **4608** | **Unplaced** | LOC111378623 | syn | **Calcineurin B-like protein 3, CBL3** | Vacuole membrane | Detection of calcium ion, acts as a calcium sensor, CBL proteins interact with CIPK serine-threonine protein kinases. Binds calcium ions, binding of a CBL protein to the regulatory NAF domain of a CIPK protein lead to the activation of the kinase in a calcium-dependent manner, mediates the activation of AKT1 by CIPK proteins (CIPK6, CIPK16, and CIPK23) in response to low potassium conditions and in the context of stomatal movement, negatively regulates the enzyme activity of MTN1 in the presence of calcium | Calcium binding |
| **5856** | **15** | LOC111410562 | syn | **NADP-dependent D-sorbitol-6-phosphate dehydrogenase-like, S6PDH** | *Not defined* | Synthesizes sorbitol-6-phosphate, a key intermediate in the synthesis of sorbitol which is a major photosynthetic product in many members of the Rosaceae family | Aldose-6-phosphate reductase (NADPH) activity |
| **6479** | **1** | PSS08062.1 | syn | **Signal peptide, CUB and EGF-like domain-containing protein** | Endoplasmic reticulum, integral component of membrane | Intracellular protein transport | *Not defined* |
| **8646** | **Unplaced** | LOC111382427 | syn | **Nuclear fusion defective 4, NFD4** | Membrane | Karyogamy, response to salt stress, required for karyogamy during female gametophyte development, when the two polar nuclei fuse to form the diploid central cell nucleus | Developmental protein |
| **9922** | **13** | LOC111407604 | syn | **6-phosphogluconate dehydrogenase decarboxylating 2 chloroplastic-like, PGD** | Cytosol, peroxisome | Male-female gamete recognition during double fertilization forming a zygote and endosperm, catalyzes the oxidative decarboxylation of 6-phosphogluconate to ribulose 5-phosphate and CO2, with concomitant reduction of NADP to NADPH, required for guided growth of the male gametophytes and interaction between the pollen tube and the ovule | NADP binding, phosphogluconate dehydrogenase (decarboxylating) activity |
| **12499** | **16** | LOC111412157 | syn | **Cellulose synthase A catalytic subunit 8, UDP-forming, CESA8** | Plasma membrane | Cell wall biogenesis-degradation, cellulose biosynthesis, catalytic subunit of cellulose synthase terminal complexes ('rosettes'), required for beta-1,4-glucan microfibril crystallization, a major mechanism of the cell wall formation, involved in the secondary cell wall formation, required for the xylem cell wall thickening | Cellulose synthase (UDP-forming) activity, metal ion binding |
| **12747** | **6** | LOC111395667 | 3'UTR | **Polyadenylate-binding protein, PABP** | Nucleus | mRNA processing, stress response, heterogeneous nuclear ribonucleoprotein (hnRNP)-protein binding the poly(A) tail of mRNA and probably involved in some steps of pre-mRNA maturation | mRNA binding |
| **1372** | **Unplaced** | XP_022858441.1 | syn | **3-oxo-Delta(4,5)-steroid 5-beta-reductase-like, or 1-4 reductase** | Cytosol | Steroid metabolic process, xylem and phloem pattern formation, involved in vascular strand development, catalyzes the stereospecific conversion of progesterone to 5-beta-pregnane-3,20-dione, can use progesterone, testosterone, 21-acetyl cortexone, 2-cyclohexenone, but-1-en-3-one, ethyl acrylate, ethylmethacrylate, cortisone and canarigenone as substrates | Delta4-3-oxosteroid 5beta-reductase activity |
| **1906** | **18** | LOC111367989 | syn | **Ribulose-phosphate 3-epimerase chloroplastic, RPE** | Chloroplast thylakoid membrane | Calvin cycle, cellular carbohydrate metabolic process, catalyzes the reversible epimerization of D-ribulose 5-phosphate to D-xylulose 5-phosphate | Metal ion binding, ribulose-phosphate 3-epimerase activity |
| **1912** | **Unplaced** | LOC111387414 | syn | **Protein HMG1/2-like** | Nucleus | Regulation of transcription, DNA-templated | DNA binding |
| **2403** | **2** | LOC111367377 | syn | **protein BI1-like** | Multi-pass membrane protein | Multi-pass membrane protein | Multi-pass membrane protein |
| **2512** | **Unplaced** | KU847999 | syn | **(+)-Neomenthol dehydrogenase, MNR** | *Not defined* | Defense response, involved in basal resistance against pathogens | (+)-neomenthol dehydrogenase activity |
| **267** | **10** | LOC111401171 | syn | **Homeobox-leucine zipper protein, ATHB-6-like** | Nucleus | negative regulation of abscisic acid-activated signaling pathway, response to water deprivation, transcription activator that may act as growth regulators in response to water deficit, interacts with the core sequence 5'-CAATTATTA-3' of promoters in response to ABA and in an ABI1-dependent manner, involved in the negative regulation of the ABA signaling pathway | DNA-binding transcription factor activity |
| **3340** | **15** | LOC111409943 | syn | **Homeobox-leucine zipper protein, ATHB-12** | Nucleus | Multicellular organism development, stress response, transcription, transcription regulation, probable transcription activator that may act as growth regulators in response to water deficit | DNA-binding transcription factor activity |
| **3480** | **12** | LOC111405311 | 3'UTR | **Probable pectate lyase 18** | Membrane | Pectin catabolic process, response to nematode, eliminative cleavage of (1-4)-alpha-D-galacturonan to give oligosaccharides with 4-deoxy-alpha-D-galact-4-enuronosyl groups at their non-reducing ends | Lyase |
| **3545** | **18** | LOC111367510 | 3'UTR | **DNA-binding protein DDB_G0278111-like** | Nucleus, cytosol | DNA repair, programmed cell death | Damaged DNA binding |
| **3656** | **Unplaced** | LOC111391952 | syn | **Abscisic acid receptor, PYL8-like** | Nucleus, Cell membrane, Cytoplasm | Receptor for abscisic acid (ABA) required for ABA-mediated responses such as stomatal closure and germination inhibition, inhibits the activity of group-A protein phosphatases type 2C (PP2Cs) in an ABA-independent manner but more efficiently when activated by ABA, confers enhanced sensitivity to ABA, can be activated by both (-)-ABA and (+)-ABA, mediates crosstalk between ABA and auxin signaling to regulate lateral root growth | Abscisic acid binding, signaling receptor activity |
| **3952** | **18** | LOC111367255 | 3'UTR | **ADP-ribosylation factor-like protein 8a** | Spindle, Lysosome membrane, Late endosome membrane | Cell cycle, defense response to virus, microbial infection, component of tomato mosaic virus (ToMV) RNA replication complexes, required for tobamovirus multiplication, especially for efficient negative-strand RNA synthesis and viral RNA capping | GTP binding |
| **4001** | **Unplaced** | PSS26580.1 | 3'UTR | **TBCC domain-containing protein** | Cytosol | Cell morphogenesis | Cell morphogenesis |
| **4108** | **Unplaced** | LOC105158075 | syn | **Nucleoside diphosphate kinase 2 chloroplastic, NDPK2** | Chloroplast | Auxin-activated signaling pathway, red, far-red light phototransduction, major role in the synthesis of nucleoside triphosphates other than ATP, the ATP gamma phosphate is transferred to the NDP beta phosphate via a ping-pong mechanism, using a phosphorylated active-site intermediate, may activate MPK3 and MPK6. May be involved in the regulation of cellular redox state and hydrogen peroxide-mediated MAP kinase signaling | ATP binding, nucleoside diphosphate kinase activity |
| **4294** | **Unplaced** | LOC111390715 | from N to D | **Protein SRG1-like** | *Not defined* | DNA integration | Metal ion binding, nucleic acid binding, oxidoreductase activity |
| **5183** | **10** | LOC111402392 | syn | **Fatty acid-binding protein-like protein, UPF0678 At1g79260** | Cytoplasm | Transport, may play a role in the intracellular transport of hydrophobic ligands | Transport |
| **539** | **Unplaced** | LOC111373345 | syn | **Aldehyde dehydrogenase family 2 member B7, mitochondrial-like** | Mitochondrion matrix | Possesses activity on acetaldehyde and glycolaldehyde in vitro | Aldehyde dehydrogenase (NAD) activity |
| **5449** | **Unplaced** | NP_567596.1 | 3'UTR | **Excitatory amino acid transporter, putative** | Mitochondrial respiratory chain complex I, vacuolar membrane | Mitochondrial respiratory chain complex I | Mitochondrial respiratory chain complex I |
| **5601** | **Unplaced** | LOC111390075 | syn | **Pollen allergen Che a 1-like** | *Not defined* | *Not defined* | *Not defined* |
| **7131** | **11** | LOC111404235 | syn | **Response to low sulfur 3-like** | *Not defined* | *Not defined* | *Not defined* |
| **749** | **7** | LOC111396744 | 5'UTR | **Pyrophosphate-fructose 6-phosphate 1-phosphotransferase subunit alpha** | Cytoplasm | Photosynthesis, sugar response, regulatory subunit of pyrophosphate--fructose 6-phosphate 1-phosphotransferase | ATP binding |
| **751** | **Unplaced** | LOC111381460 | syn | **Probable serine/threonine-protein kinase, PBL8** | Cell membrane | May be involved in plant defense signaling | ATP binding |
| **754** | **6** | LOC111396063 | syn | **S-adenosylmethionine synthase 1** | Cytoplasm | Cellular response to iron ion, ethylene biosynthetic process, catalyzes the formation of S-adenosylmethionine from methionine and ATP, the reaction comprises two steps that are both catalyzed by the same enzyme: formation of S-adenosylmethionine (AdoMet) and triphosphate, and subsequent hydrolysis of the triphosphate | ATP binding, methionine adenosyltransferase activity |
| **761** | **6** | LOC111395620 | syn | **Protein detoxification 18-like** | Integral component of membrane | Drug transmembrane transporter activity | Drug transmembrane transporter activity |
| **8198** | **10** | LOC111401439 | from Q to P | **Putative glucose-6-phosphate 1-epimerase** | Chloroplast stroma, apoplast | Carbohydrate metabolic process | Isomerase, carbohydrate binding |
| **9524** | **13** | PSS17738.1 | syn | **NADPH oxidase** | Integral component of membrane | Superoxide anion generation | Superoxide-generating NADPH oxidase activity |
| **9722** | **Unplaced** | LOC105160273 | syn | **Tetratricopeptide repeat protein 5** | Cytoplasm | Posttranscriptional gene silencing | DNA binding |

* As reported in Unver et al. (2017).
