# Supplementary material for: EST–SNP Study of Olea europaea L. Uncovers Functional Polymorphisms between Cultivated and Wild Olives
Source: Genes (Basel). 2020 Aug 10;11(8):916. doi: 10.3390/genes11080916 (PMC7465833; doi:10.3390/genes11080916)
Supplement: Supplementary file 1 [file genes-11-00916-s001.zip › Table_S6.docx]

**Table S6.** List of the biological processes detected for the 124 genes differentiating olive cultivars from wilds and subsp. *guanchica* samples. The analysis was performed by PANTHER Overrepresentation Test.

| **GO biological processes** | ***Arabidopsis thaliana***  **REFLIST (27502)** | **upload_1** | **upload_1 (expected)** | **upload_1 (over/under)** | **upload_1 (fold Enrichment)** | **upload_1 (raw P-value)** |
| --- | --- | --- | --- | --- | --- | --- |
| Biological process (GO:0008150) | 22541 | 15 | 13.93 | + | 1.08 | 7.54E-01 |
| Cellular process (GO:0009987) | 11422 | 12 | 7.06 | + | 1.7 | 2.38E-02 |
| Response to stimulus (GO:0050896) | 6214 | 11 | 3.84 | + | 2.86 | 2.44E-04 |
| Cellular Metabolic process (GO:0044237) | 7949 | 8 | 4.91 | + | 1.63 | 1.11E-01 |
| Organic substance metabolic process (GO:0071704) | 8341 | 8 | 5.16 | + | 1.55 | 1.84E-01 |
| Metabolic process (GO:0008152) | 9472 | 8 | 5.85 | + | 1.37 | 3.10E-01 |
| Response to stress (GO:0006950) | 3552 | 6 | 2.2 | + | 2.73 | 1.62E-02 |
| Macromolecule metabolic process (GO:0043170) | 5642 | 6 | 3.49 | + | 1.72 | 1.37E-01 |
| Primary metabolic process (GO:0044238) | 7610 | 6 | 4.7 | + | 1.28 | 5.87E-01 |
| Cellular response to stimulus (GO:0051716) | 2697 | 5 | 1.67 | + | 3 | 2.06E-02 |
| Response to chemical (GO:0042221) | 2874 | 5 | 1.78 | + | 2.81 | 2.64E-02 |
| Organonitrogen compound metabolic process (GO:1901564) | 4857 | 5 | 3 | + | 1.67 | 2.04E-01 |
| Biological regulation (GO:0065007) | 6055 | 5 | 3.74 | + | 1.34 | 5.56E-01 |
| Nitrogen compound metabolic process (GO:0006807) | 6477 | 5 | 4 | + | 1.25 | 5.70E-01 |
| Drug metabolic process (GO:0017144) | 707 | 4 | 0.44 | + | 9.15 | 8.04E-04 |
| Organic substance biosynthetic process (GO:1901576) | 2873 | 4 | 1.78 | + | 2.25 | 9.40E-02 |
| Biosynthetic process (GO:0009058) | 3001 | 4 | 1.86 | + | 2.16 | 1.06E-01 |
| Cellular component organization or biogenesis (GO:0071840) | 3089 | 4 | 1.91 | + | 2.09 | 1.15E-01 |
| Cellular protein metabolic process (GO:0044267) | 3270 | 4 | 2.02 | + | 1.98 | 1.35E-01 |
| Protein metabolic process (GO:0019538) | 3640 | 4 | 2.25 | + | 1.78 | 2.69E-01 |
| Cellular Macromolecule metabolic process (GO:0044260) | 4335 | 4 | 2.68 | + | 1.49 | 3.29E-01 |
| Response to inorganic substance (GO:0010035) | 935 | 3 | 0.58 | + | 5.19 | 1.88E-02 |
| Response to external biotic stimulus (GO:0043207) | 1253 | 3 | 0.77 | + | 3.87 | 4.00E-02 |
| Response to other organism (GO:0051707) | 1253 | 3 | 0.77 | + | 3.87 | 4.00E-02 |
| Response to biotic stimulus (GO:0009607) | 1266 | 3 | 0.78 | + | 3.83 | 4.10E-02 |
| Defense response (GO:0006952) | 1513 | 3 | 0.94 | + | 3.21 | 6.37E-02 |
| Response to external stimulus (GO:0009605) | 1659 | 3 | 1.03 | + | 2.93 | 7.93E-02 |
| Response to hormone (GO:0009725) | 1728 | 3 | 1.07 | + | 2.81 | 8.73E-02 |
| Response to endogenous stimulus (GO:0009719) | 1745 | 3 | 1.08 | + | 2.78 | 8.93E-02 |
| Multi-organism process (GO:0051704) | 1759 | 3 | 1.09 | + | 2.76 | 9.10E-02 |
| Response to organic substance (GO:0010033) | 2026 | 3 | 1.25 | + | 2.4 | 1.26E-01 |
| Response to abiotic stimulus (GO:0009628) | 2097 | 3 | 1.3 | + | 2.31 | 1.36E-01 |
| Protein modification process (GO:0036211) | 2328 | 3 | 1.44 | + | 2.08 | 1.70E-01 |
| Cellular protein modification process (GO:0006464) | 2328 | 3 | 1.44 | + | 2.08 | 1.70E-01 |
| Cellular component organization (GO:0016043) | 2738 | 3 | 1.69 | + | 1.77 | 2.36E-01 |
| Macromolecule modification (GO:0043412) | 2741 | 3 | 1.69 | + | 1.77 | 2.37E-01 |
| Cellular biosynthetic process (GO:0044249) | 2758 | 3 | 1.7 | + | 1.76 | 2.40E-01 |
| Cellular nitrogen compound metabolic process (GO:0034641) | 3138 | 3 | 1.94 | + | 1.55 | 4.33E-01 |
| Regulation of cellular process (GO:0050794) | 4744 | 3 | 2.93 | + | 1.02 | 1.00E+00 |
| Regulation of biological process (GO:0050789) | 5383 | 3 | 3.33 | - | 0.9 | 1.00E+00 |
| Response to virus (GO:0009615) | 80 | 2 | 0.05 | + | 40.44 | 1.16E-03 |
| Pectin metabolic process (GO:0045488) | 148 | 2 | 0.09 | + | 21.86 | 3.80E-03 |
| Galacturonan metabolic process (GO:0010393) | 149 | 2 | 0.09 | + | 21.71 | 3.85E-03 |
| Polysaccharide metabolic process (GO:0005976) | 443 | 2 | 0.27 | + | 7.3 | 3.02E-02 |
| Response to metal ion (GO:0010038) | 476 | 2 | 0.29 | + | 6.8 | 3.45E-02 |
| Response to salt stress (GO:0009651) | 585 | 2 | 0.36 | + | 5.53 | 5.00E-02 |
| Response to osmotic stress (GO:0006970) | 660 | 2 | 0.41 | + | 4.9 | 6.19E-02 |
| Protein phosphorylation (GO:0006468) | 963 | 2 | 0.6 | + | 3.36 | 1.18E-01 |
| Carbohydrate metabolic process (GO:0005975) | 1025 | 2 | 0.63 | + | 3.16 | 1.31E-01 |
| Cellular response to chemical stimulus (GO:0070887) | 1166 | 2 | 0.72 | + | 2.77 | 1.61E-01 |
| Regulation of biological quality (GO:0065008) | 1192 | 2 | 0.74 | + | 2.71 | 1.67E-01 |
| Response to acid chemical (GO:0001101) | 1198 | 2 | 0.74 | + | 2.7 | 1.68E-01 |
| Cellular component biogenesis (GO:0044085) | 1246 | 2 | 0.77 | + | 2.6 | 1.79E-01 |
| Phosphorylation (GO:0016310) | 1308 | 2 | 0.81 | + | 2.47 | 1.93E-01 |
| Macromolecule biosynthetic process (GO:0009059) | 1361 | 2 | 0.84 | + | 2.38 | 2.05E-01 |
| Cellular nitrogen compound biosynthetic process (GO:0044271) | 1366 | 2 | 0.84 | + | 2.37 | 2.06E-01 |
| Organonitrogen compound biosynthetic process (GO:1901566) | 1500 | 2 | 0.93 | + | 2.16 | 2.37E-01 |
| Organelle organization (GO:0006996) | 1613 | 2 | 1 | + | 2.01 | 2.63E-01 |
| Response to oxygen-containing compound (GO:1901700) | 1619 | 2 | 1 | + | 2 | 2.65E-01 |
| Signal transduction (GO:0007165) | 1750 | 2 | 1.08 | + | 1.85 | 2.95E-01 |
| Signaling (GO:0023052) | 1779 | 2 | 1.1 | + | 1.82 | 3.02E-01 |
| Phosphate-containing compound metabolic process (GO:0006796) | 1900 | 2 | 1.17 | + | 1.7 | 3.31E-01 |
| Phosphorus metabolic process (GO:0006793) | 1943 | 2 | 1.2 | + | 1.67 | 3.41E-01 |
| Cell communication (GO:0007154) | 2009 | 2 | 1.24 | + | 1.61 | 3.56E-01 |
| Unclassified (UNCLASSIFIED) | 4961 | 2 | 3.07 | - | 0.65 | 7.54E-01 |
| Cellular response to gravity (GO:0071258) | 3 | 1 | 0 | + | > 100 | 2.47E-03 |
| S-adenosylmethionine biosynthetic process (GO:0006556) | 4 | 1 | 0 | + | > 100 | 3.09E-03 |
| Spermine biosynthetic process (GO:0006597) | 6 | 1 | 0 | + | > 100 | 4.32E-03 |
| Endoplasmic reticulum tubular network organization (GO:0071786) | 7 | 1 | 0 | + | > 100 | 4.93E-03 |
| Detection of calcium ion (GO:0005513) | 7 | 1 | 0 | + | > 100 | 4.93E-03 |
| S-adenosylmethionine metabolic process (GO:0046500) | 8 | 1 | 0 | + | > 100 | 5.55E-03 |
| Spermidine biosynthetic process (GO:0008295) | 8 | 1 | 0 | + | > 100 | 5.55E-03 |
| Spermine metabolic process (GO:0008215) | 8 | 1 | 0 | + | > 100 | 5.55E-03 |
| Spermidine metabolic process (GO:0008216) | 12 | 1 | 0.01 | + | > 100 | 8.00E-03 |
| Response to calcium ion (GO:0051592) | 16 | 1 | 0.01 | + | > 100 | 1.05E-02 |
| Polyamine biosynthetic process (GO:0006596) | 18 | 1 | 0.01 | + | 89.88 | 1.17E-02 |
| Potassium ion homeostasis (GO:0055075) | 19 | 1 | 0.01 | + | 85.15 | 1.23E-02 |
| Endoplasmic reticulum organization (GO:0007029) | 21 | 1 | 0.01 | + | 77.04 | 1.35E-02 |
| Detection of chemical stimulus (GO:0009593) | 23 | 1 | 0.01 | + | 70.34 | 1.47E-02 |
| Polyamine metabolic process (GO:0006595) | 25 | 1 | 0.02 | + | 64.71 | 1.59E-02 |
| Olefin biosynthetic process (GO:1900674) | 26 | 1 | 0.02 | + | 62.22 | 1.66E-02 |
| Olefin metabolic process (GO:1900673) | 26 | 1 | 0.02 | + | 62.22 | 1.66E-02 |
| Ethylene biosynthetic process (GO:0009693) | 26 | 1 | 0.02 | + | 62.22 | 1.66E-02 |
| Ethylene metabolic process (GO:0009692) | 26 | 1 | 0.02 | + | 62.22 | 1.66E-02 |
| Alkene biosynthetic process (GO:0043450) | 26 | 1 | 0.02 | + | 62.22 | 1.66E-02 |
| Cellular alkene metabolic process (GO:0043449) | 26 | 1 | 0.02 | + | 62.22 | 1.66E-02 |
| Cellular response to iron ion (GO:0071281) | 33 | 1 | 0.02 | + | 49.02 | 2.08E-02 |
| One-carbon metabolic process (GO:0006730) | 36 | 1 | 0.02 | + | 44.94 | 2.26E-02 |
| Regulation of jasmonic acid mediated signaling pathway (GO:2000022) | 38 | 1 | 0.02 | + | 42.57 | 2.38E-02 |
| Defense response to virus (GO:0051607) | 43 | 1 | 0.03 | + | 37.62 | 2.68E-02 |
| Protein-chromophore linkage (GO:0018298) | 44 | 1 | 0.03 | + | 36.77 | 2.74E-02 |
| Pectin biosynthetic process (GO:0045489) | 46 | 1 | 0.03 | + | 35.17 | 2.86E-02 |
| Amine biosynthetic process (GO:0009309) | 46 | 1 | 0.03 | + | 35.17 | 2.86E-02 |
| Cellular biogenic amine biosynthetic process (GO:0042401) | 46 | 1 | 0.03 | + | 35.17 | 2.86E-02 |
| Ammonium ion metabolic process (GO:0097164) | 50 | 1 | 0.03 | + | 32.36 | 3.11E-02 |
| Response to iron ion (GO:0010039) | 51 | 1 | 0.03 | + | 31.72 | 3.17E-02 |
| Cellular response to metal ion (GO:0071248) | 53 | 1 | 0.03 | + | 30.52 | 3.28E-02 |
| Cellular biogenic amine metabolic process (GO:0006576) | 61 | 1 | 0.04 | + | 26.52 | 3.76E-02 |
| Cellular amine metabolic process (GO:0044106) | 61 | 1 | 0.04 | + | 26.52 | 3.76E-02 |
| Immune effector process (GO:0002252) | 62 | 1 | 0.04 | + | 26.09 | 3.82E-02 |
| Jasmonic acid mediated signaling pathway (GO:0009867) | 65 | 1 | 0.04 | + | 24.89 | 4.00E-02 |
| Cellular response to inorganic substance (GO:0071241) | 67 | 1 | 0.04 | + | 24.15 | 4.12E-02 |
| Cellular response to jasmonic acid stimulus (GO:0071395) | 69 | 1 | 0.04 | + | 23.45 | 4.24E-02 |
| Response to nematode (GO:0009624) | 76 | 1 | 0.05 | + | 21.29 | 4.65E-02 |
| Monovalent inorganic cation homeostasis (GO:0055067) | 83 | 1 | 0.05 | + | 19.49 | 5.07E-02 |
| Response to gravity (GO:0009629) | 86 | 1 | 0.05 | + | 18.81 | 5.24E-02 |
| Detection of stimulus (GO:0051606) | 90 | 1 | 0.06 | + | 17.98 | 5.48E-02 |
| Pectin catabolic process (GO:0045490) | 96 | 1 | 0.06 | + | 16.85 | 5.83E-02 |
| Endomembrane system organization (GO:0010256) | 96 | 1 | 0.06 | + | 16.85 | 5.83E-02 |
| Amine metabolic process (GO:0009308) | 113 | 1 | 0.07 | + | 14.32 | 6.82E-02 |
| Chromosome segregation (GO:0007059) | 119 | 1 | 0.07 | + | 13.59 | 7.16E-02 |
| Microtubule cytoskeleton organization (GO:0000226) | 137 | 1 | 0.08 | + | 11.81 | 8.19E-02 |
| Sulfur compound biosynthetic process (GO:0044272) | 153 | 1 | 0.09 | + | 10.57 | 9.10E-02 |
| Metal ion homeostasis (GO:0055065) | 156 | 1 | 0.1 | + | 10.37 | 9.27E-02 |
| Polysaccharide catabolic process (GO:0000272) | 183 | 1 | 0.11 | + | 8.84 | 1.08E-01 |
| Hormone biosynthetic process (GO:0042446) | 183 | 1 | 0.11 | + | 8.84 | 1.08E-01 |
| Polysaccharide biosynthetic process (GO:0000271) | 201 | 1 | 0.12 | + | 8.05 | 1.18E-01 |
| Cellular response to environmental stimulus (GO:0104004) | 202 | 1 | 0.12 | + | 8.01 | 1.18E-01 |
| Cellular response to abiotic stimulus (GO:0071214) | 202 | 1 | 0.12 | + | 8.01 | 1.18E-01 |
| Microtubule-based process (GO:0007017) | 205 | 1 | 0.13 | + | 7.89 | 1.20E-01 |
| Response to jasmonic acid (GO:0009753) | 213 | 1 | 0.13 | + | 7.6 | 1.24E-01 |
| Cation homeostasis (GO:0055080) | 215 | 1 | 0.13 | + | 7.52 | 1.25E-01 |
| Response to wounding (GO:0009611) | 215 | 1 | 0.13 | + | 7.52 | 1.25E-01 |
| Coenzyme biosynthetic process (GO:0009108) | 222 | 1 | 0.14 | + | 7.29 | 1.29E-01 |
| Cell wall biogenesis (GO:0042546) | 225 | 1 | 0.14 | + | 7.19 | 1.31E-01 |
| Inorganic ion homeostasis (GO:0098771) | 228 | 1 | 0.14 | + | 7.1 | 1.32E-01 |
| Cytoskeleton organization (GO:0007010) | 240 | 1 | 0.15 | + | 6.74 | 1.39E-01 |
| Response to cytokinin (GO:0009735) | 245 | 1 | 0.15 | + | 6.6 | 1.42E-01 |
| Drug catabolic process (GO:0042737) | 248 | 1 | 0.15 | + | 6.52 | 1.43E-01 |
| Regulation of defense response (GO:0031347) | 252 | 1 | 0.16 | + | 6.42 | 1.45E-01 |
| Hormone metabolic process (GO:0042445) | 259 | 1 | 0.16 | + | 6.25 | 1.49E-01 |
| Mitotic cell cycle (GO:0000278) | 260 | 1 | 0.16 | + | 6.22 | 1.50E-01 |
| Ion homeostasis (GO:0050801) | 260 | 1 | 0.16 | + | 6.22 | 1.50E-01 |
| Cofactor biosynthetic process (GO:0051188) | 284 | 1 | 0.18 | + | 5.7 | 1.62E-01 |
| Carbohydrate catabolic process (GO:0016052) | 289 | 1 | 0.18 | + | 5.6 | 1.65E-01 |
| Regulation of signal transduction (GO:0009966) | 297 | 1 | 0.18 | + | 5.45 | 1.69E-01 |
| DNA repair (GO:0006281) | 304 | 1 | 0.19 | + | 5.32 | 1.73E-01 |
| Regulation of signaling (GO:0023051) | 304 | 1 | 0.19 | + | 5.32 | 1.73E-01 |
| Regulation of cell communication (GO:0010646) | 305 | 1 | 0.19 | + | 5.3 | 1.73E-01 |
| Coenzyme metabolic process (GO:0006732) | 316 | 1 | 0.2 | + | 5.12 | 1.79E-01 |
| Cell division (GO:0051301) | 325 | 1 | 0.2 | + | 4.98 | 1.83E-01 |
| Cellular response to DNA damage stimulus (GO:0006974) | 335 | 1 | 0.21 | + | 4.83 | 1.89E-01 |
| Carbohydrate biosynthetic process (GO:0016051) | 340 | 1 | 0.21 | + | 4.76 | 1.91E-01 |
| Response to cadmium ion (GO:0046686) | 343 | 1 | 0.21 | + | 4.72 | 1.93E-01 |
| Response to water deprivation (GO:0009414) | 346 | 1 | 0.21 | + | 4.68 | 1.94E-01 |
| Response to water (GO:0009415) | 353 | 1 | 0.22 | + | 4.58 | 1.98E-01 |
| Regulation of response to stress (GO:0080134) | 361 | 1 | 0.22 | + | 4.48 | 2.02E-01 |
| Immune system process (GO:0002376) | 373 | 1 | 0.23 | + | 4.34 | 2.08E-01 |
| Chemical homeostasis (GO:0048878) | 379 | 1 | 0.23 | + | 4.27 | 2.11E-01 |
| Sulfur compound metabolic process (GO:0006790) | 390 | 1 | 0.24 | + | 4.15 | 2.16E-01 |
| Ribosome biogenesis (GO:0042254) | 396 | 1 | 0.24 | + | 4.09 | 2.19E-01 |
| Regulation of hormone levels (GO:0010817) | 409 | 1 | 0.25 | + | 3.96 | 2.25E-01 |
| Cellular response to acid chemical (GO:0071229) | 449 | 1 | 0.28 | + | 3.6 | 2.44E-01 |
| Ribonucleoprotein complex biogenesis (GO:0022613) | 481 | 1 | 0.3 | + | 3.36 | 2.60E-01 |
| Positive regulation of transcription, DNA-templated (GO:0045893) | 495 | 1 | 0.31 | + | 3.27 | 2.66E-01 |
| Positive regulation of RNA biosynthetic process (GO:1902680) | 497 | 1 | 0.31 | + | 3.26 | 2.67E-01 |
| Positive regulation of nucleic acid-templated transcription (GO:1903508) | 497 | 1 | 0.31 | + | 3.26 | 2.67E-01 |
| Cell wall organization (GO:0071555) | 504 | 1 | 0.31 | + | 3.21 | 2.70E-01 |
| DNA metabolic process (GO:0006259) | 515 | 1 | 0.32 | + | 3.14 | 2.75E-01 |
| Positive regulation of RNA metabolic process (GO:0051254) | 518 | 1 | 0.32 | + | 3.12 | 2.77E-01 |
| Cell cycle (GO:0007049) | 529 | 1 | 0.33 | + | 3.06 | 2.82E-01 |
| Positive regulation of macromolecule biosynthetic process (GO:0010557) | 536 | 1 | 0.33 | + | 3.02 | 2.85E-01 |
| External encapsulating structure organization (GO:0045229) | 541 | 1 | 0.33 | + | 2.99 | 2.87E-01 |
| Positive regulation of nucleobase-containing compound metabolic process (GO:0045935) | 548 | 1 | 0.34 | + | 2.95 | 2.90E-01 |
| Positive regulation of gene expression (GO:0010628) | 554 | 1 | 0.34 | + | 2.92 | 2.93E-01 |
| Positive regulation of cellular biosynthetic process (GO:0031328) | 567 | 1 | 0.35 | + | 2.85 | 2.99E-01 |
| Homeostatic process (GO:0042592) | 571 | 1 | 0.35 | + | 2.83 | 3.00E-01 |
| Response to abscisic acid (GO:0009737) | 578 | 1 | 0.36 | + | 2.8 | 3.03E-01 |
| Positive regulation of biosynthetic process (GO:0009891) | 580 | 1 | 0.36 | + | 2.79 | 3.04E-01 |
| Cofactor metabolic process (GO:0051186) | 580 | 1 | 0.36 | + | 2.79 | 3.04E-01 |
| Response to alcohol (GO:0097305) | 582 | 1 | 0.36 | + | 2.78 | 3.05E-01 |
| Translation (GO:0006412) | 588 | 1 | 0.36 | + | 2.75 | 3.08E-01 |
| Peptide biosynthetic process (GO:0043043) | 593 | 1 | 0.37 | + | 2.73 | 3.10E-01 |
| Intracellular signal transduction (GO:0035556) | 600 | 1 | 0.37 | + | 2.7 | 3.13E-01 |
| Cellular response to oxygen-containing compound (GO:1901701) | 633 | 1 | 0.39 | + | 2.56 | 3.27E-01 |
| Cell wall organization or biogenesis (GO:0071554) | 657 | 1 | 0.41 | + | 2.46 | 3.37E-01 |
| Amide biosynthetic process (GO:0043604) | 664 | 1 | 0.41 | + | 2.44 | 3.40E-01 |
| Peptide metabolic process (GO:0006518) | 671 | 1 | 0.41 | + | 2.41 | 3.43E-01 |
| Regulation of response to stimulus (GO:0048583) | 683 | 1 | 0.42 | + | 2.37 | 3.48E-01 |
| Positive regulation of nitrogen compound metabolic process (GO:0051173) | 737 | 1 | 0.46 | + | 2.2 | 3.70E-01 |
| Positive regulation of macromolecule metabolic process (GO:0010604) | 752 | 1 | 0.46 | + | 2.15 | 3.76E-01 |
| Hormone-mediated signaling pathway (GO:0009755) | 770 | 1 | 0.48 | + | 2.1 | 3.83E-01 |
| Positive regulation of cellular metabolic process (GO:0031325) | 779 | 1 | 0.48 | + | 2.08 | 3.87E-01 |
| Response to lipid (GO:0033993) | 787 | 1 | 0.49 | + | 2.06 | 3.90E-01 |
| Cellular amide metabolic process (GO:0043603) | 808 | 1 | 0.5 | + | 2 | 3.98E-01 |
| Positive regulation of metabolic process (GO:0009893) | 827 | 1 | 0.51 | + | 1.96 | 4.05E-01 |
| Cellular response to hormone stimulus (GO:0032870) | 881 | 1 | 0.54 | + | 1.84 | 4.25E-01 |
| Cellular response to endogenous stimulus (GO:0071495) | 898 | 1 | 0.56 | + | 1.8 | 4.32E-01 |
| Cellular response to stress (GO:0033554) | 943 | 1 | 0.58 | + | 1.72 | 4.48E-01 |
| Defense response to other organism (GO:0098542) | 952 | 1 | 0.59 | + | 1.7 | 4.51E-01 |
| Macromolecule catabolic process (GO:0009057) | 961 | 1 | 0.59 | + | 1.68 | 4.54E-01 |
| Cellular response to organic substance (GO:0071310) | 991 | 1 | 0.61 | + | 1.63 | 4.64E-01 |
| Positive regulation of cellular process (GO:0048522) | 1033 | 1 | 0.64 | + | 1.57 | 4.79E-01 |
| Positive regulation of biological process (GO:0048518) | 1289 | 1 | 0.8 | + | 1.26 | 5.58E-01 |
| Cellular macromolecule biosynthetic process (GO:0034645) | 1306 | 1 | 0.81 | + | 1.24 | 5.63E-01 |
| Cellular catabolic process (GO:0044248) | 1471 | 1 | 0.91 | + | 1.1 | 6.07E-01 |
| Organic substance catabolic process (GO:1901575) | 1553 | 1 | 0.96 | + | 1.04 | 1.00E+00 |
| Gene expression (GO:0010467) | 1605 | 1 | 0.99 | + | 1.01 | 1.00E+00 |
| Small molecule metabolic process (GO:0044281) | 1745 | 1 | 1.08 | - | 0.93 | 1.00E+00 |
| Catabolic process (GO:0009056) | 1772 | 1 | 1.1 | - | 0.91 | 1.00E+00 |
| Nucleic acid metabolic process (GO:0090304) | 1776 | 1 | 1.1 | - | 0.91 | 1.00E+00 |
| Regulation of transcription, DNA-templated (GO:0006355) | 2125 | 1 | 1.31 | - | 0.76 | 1.00E+00 |
| Regulation of RNA biosynthetic process (GO:2001141) | 2126 | 1 | 1.31 | - | 0.76 | 1.00E+00 |
| Regulation of nucleic acid-templated transcription (GO:1903506) | 2126 | 1 | 1.31 | - | 0.76 | 1.00E+00 |
| Regulation of RNA metabolic process (GO:0051252) | 2203 | 1 | 1.36 | - | 0.73 | 1.00E+00 |
| Nucleobase-containing compound metabolic process (GO:0006139) | 2272 | 1 | 1.4 | - | 0.71 | 1.00E+00 |
| Regulation of nucleobase-containing compound metabolic process (GO:0019219) | 2290 | 1 | 1.42 | - | 0.71 | 1.00E+00 |
| Regulation of cellular macromolecule biosynthetic process (GO:2000112) | 2326 | 1 | 1.44 | - | 0.7 | 1.00E+00 |
| Regulation of macromolecule biosynthetic process (GO:0010556) | 2335 | 1 | 1.44 | - | 0.69 | 1.00E+00 |
| Regulation of cellular biosynthetic process (GO:0031326) | 2425 | 1 | 1.5 | - | 0.67 | 1.00E+00 |
| Regulation of biosynthetic process (GO:0009889) | 2449 | 1 | 1.51 | - | 0.66 | 1.00E+00 |
| Regulation of gene expression (GO:0010468) | 2578 | 1 | 1.59 | - | 0.63 | 1.00E+00 |
| Heterocycle metabolic process (GO:0046483) | 2584 | 1 | 1.6 | - | 0.63 | 1.00E+00 |
| Multicellular organism development (GO:0007275) | 2722 | 1 | 1.68 | - | 0.59 | 1.00E+00 |
| Cellular aromatic compound metabolic process (GO:0006725) | 2748 | 1 | 1.7 | - | 0.59 | 1.00E+00 |
| Regulation of nitrogen compound metabolic process (GO:0051171) | 2758 | 1 | 1.7 | - | 0.59 | 1.00E+00 |
| Regulation of primary metabolic process (GO:0080090) | 2825 | 1 | 1.75 | - | 0.57 | 1.00E+00 |
| Organic cyclic compound metabolic process (GO:1901360) | 2867 | 1 | 1.77 | - | 0.56 | 1.00E+00 |
| Regulation of cellular metabolic process (GO:0031323) | 2934 | 1 | 1.81 | - | 0.55 | 1.00E+00 |
| Multicellular organismal process (GO:0032501) | 2950 | 1 | 1.82 | - | 0.55 | 1.00E+00 |
| Regulation of macromolecule metabolic process (GO:0060255) | 2961 | 1 | 1.83 | - | 0.55 | 1.00E+00 |
| Anatomical structure development (GO:0048856) | 3052 | 1 | 1.89 | - | 0.53 | 1.00E+00 |
| Regulation of metabolic process (GO:0019222) | 3206 | 1 | 1.98 | - | 0.5 | 7.12E-01 |
| Developmental process (GO:0032502) | 3233 | 1 | 2 | - | 0.5 | 7.12E-01 |
