# Supplementary material for: EST–SNP Study of Olea europaea L. Uncovers Functional Polymorphisms between Cultivated and Wild Olives
Source: Genes (Basel). 2020 Aug 10;11(8):916. doi: 10.3390/genes11080916 (PMC7465833; doi:10.3390/genes11080916)
Supplement: Supplementary file 1 [file genes-11-00916-s001.zip › Table_S7.docx]

**Table S7.** List of all cellular components detected for the 124 genes differentiating olive cultivars from wilds and subsp. *guanchica* samples. The analysis was performed by PANTHER Overrepresentation Test.

| **GO cellular components** | ***Arabidopsis thaliana***  **REFLIST (27502)** | **upload_1** | **upload_1 (expected)** | **upload_1 (over/under)** | **upload_1 (fold Enrichment)** | **upload_1 (raw P-value)** |
| --- | --- | --- | --- | --- | --- | --- |
| Membrane (GO:0016020) | 8172 | 11 | 5.05 | + | 2.18 | 2.99E-03 |
| Plasma membrane (GO:0005886) | 3505 | 8 | 2.17 | + | 3.69 | 5.82E-04 |
| Cell periphery (GO:0071944) | 4157 | 8 | 2.57 | + | 3.11 | 1.84E-03 |
| Bounding membrane of organelle (GO:0098588) | 1300 | 5 | 0.8 | + | 6.22 | 9.13E-04 |
| Organelle membrane (GO:0031090) | 1866 | 5 | 1.15 | + | 4.33 | 4.48E-03 |
| Cytosol (GO:0005829) | 2226 | 5 | 1.38 | + | 3.63 | 9.44E-03 |
| Vacuolar membrane (GO:0005774) | 634 | 4 | 0.39 | + | 10.21 | 5.36E-04 |
| Vacuolar part (GO:0044437) | 636 | 4 | 0.39 | + | 10.17 | 5.42E-04 |
| Whole membrane (GO:0098805) | 980 | 4 | 0.61 | + | 6.6 | 2.66E-03 |
| Vacuole (GO:0005773) | 1088 | 4 | 0.67 | + | 5.95 | 3.88E-03 |
| Cell wall (GO:0005618) | 777 | 3 | 0.48 | + | 6.25 | 1.15E-02 |
| External encapsulating structure (GO:0030312) | 779 | 3 | 0.48 | + | 6.23 | 1.15E-02 |
| Plasmodesma (GO:0009506) | 1008 | 3 | 0.62 | + | 4.81 | 2.29E-02 |
| Symplast (GO:0055044) | 1008 | 3 | 0.62 | + | 4.81 | 2.29E-02 |
| Cell-cell junction (GO:0005911) | 1010 | 3 | 0.62 | + | 4.81 | 2.30E-02 |
| Cell junction (GO:0030054) | 1010 | 3 | 0.62 | + | 4.81 | 2.30E-02 |
| Golgi apparatus (GO:0005794) | 1183 | 3 | 0.73 | + | 4.1 | 3.46E-02 |
| Microtubule cytoskeleton (GO:0015630) | 275 | 2 | 0.17 | + | 11.77 | 1.24E-02 |
| Cytoskeletal part (GO:0044430) | 314 | 2 | 0.19 | + | 10.3 | 1.60E-02 |
| Cytoskeleton (GO:0005856) | 394 | 2 | 0.24 | + | 8.21 | 2.44E-02 |
| Integral component of cytoplasmic side of endoplasmic reticulum membrane (GO:0071458) | 11 | 1 | 0.01 | + | > 100 | 7.39E-03 |
| Endoplasmic reticulum tubular network (GO:0071782) | 11 | 1 | 0.01 | + | > 100 | 7.39E-03 |
| Cytoplasmic side of endoplasmic reticulum membrane (GO:0098554) | 11 | 1 | 0.01 | + | > 100 | 7.39E-03 |
| Tubulin complex (GO:0045298) | 13 | 1 | 0.01 | + | > 100 | 8.62E-03 |
| Lysosomal membrane (GO:0005765) | 15 | 1 | 0.01 | + | > 100 | 9.84E-03 |
| Lytic vacuole membrane (GO:0098852) | 15 | 1 | 0.01 | + | > 100 | 9.84E-03 |
| Lysosome (GO:0005764) | 50 | 1 | 0.03 | + | 32.36 | 3.11E-02 |
| Lytic vacuole (GO:0000323) | 53 | 1 | 0.03 | + | 30.52 | 3.28E-02 |
| Late endosome membrane (GO:0031902) | 63 | 1 | 0.04 | + | 25.68 | 3.88E-02 |
| Mitochondrial respiratory chain complex I (GO:0005747) | 68 | 1 | 0.04 | + | 23.79 | 4.18E-02 |
| Integral component of endoplasmic reticulum membrane (GO:0030176) | 70 | 1 | 0.04 | + | 23.11 | 4.30E-02 |
| Respiratory chain complex I (GO:0045271) | 71 | 1 | 0.04 | + | 22.79 | 4.36E-02 |
| NADH dehydrogenase complex (GO:0030964) | 72 | 1 | 0.04 | + | 22.47 | 4.42E-02 |
| Intrinsic component of endoplasmic reticulum membrane (GO:0031227) | 72 | 1 | 0.04 | + | 22.47 | 4.42E-02 |
| Spindle (GO:0005819) | 79 | 1 | 0.05 | + | 20.48 | 4.83E-02 |
