# Supplementary material for: EST–SNP Study of Olea europaea L. Uncovers Functional Polymorphisms between Cultivated and Wild Olives
Source: Genes (Basel). 2020 Aug 10;11(8):916. doi: 10.3390/genes11080916 (PMC7465833; doi:10.3390/genes11080916)
Supplement: Supplementary file 1 [file genes-11-00916-s001.zip › Table_S8.docx]

**Table S8.** List of all molecular functions detected for the 124 genes differentiating olive cultivars from wilds and subsp. *guanchica* samples. The analysis was performed by PANTHER Overrepresentation Test.

| **GO molecular function** | ***Arabidopsis thaliana* REFLIST (27502)** | **upload_1** | **upload_1 (expected)** | **upload_1 (over/under)** | **upload_1 (fold Enrichment)** | **upload_1 (raw P-value)** |
| --- | --- | --- | --- | --- | --- | --- |
| Molecular_function (GO:0003674) | 22419 | 15 | 13.86 | + | 1.08 | 7.54E-01 |
| Binding (GO:0005488) | 11083 | 11 | 6.85 | + | 1.61 | 4.84E-02 |
| Ion binding (GO:0043167) | 5438 | 8 | 3.36 | + | 2.38 | 1.02E-02 |
| Heterocyclic compound binding (GO:1901363) | 6460 | 8 | 3.99 | + | 2 | 3.89E-02 |
| Organic cyclic compound binding (GO:0097159) | 6483 | 8 | 4.01 | + | 2 | 3.93E-02 |
| Catalytic activity (GO:0003824) | 9288 | 8 | 5.74 | + | 1.39 | 3.05E-01 |
| Purine ribonucleoside triphosphate binding (GO:0035639) | 2198 | 6 | 1.36 | + | 4.42 | 1.50E-03 |
| Purine ribonucleotide binding (GO:0032555) | 2278 | 6 | 1.41 | + | 4.26 | 1.80E-03 |
| Purine nucleotide binding (GO:0017076) | 2286 | 6 | 1.41 | + | 4.25 | 1.83E-03 |
| Ribonucleotide binding (GO:0032553) | 2299 | 6 | 1.42 | + | 4.22 | 1.89E-03 |
| Carbohydrate derivative binding (GO:0097367) | 2335 | 6 | 1.44 | + | 4.16 | 2.05E-03 |
| Nucleotide binding (GO:0000166) | 2724 | 6 | 1.68 | + | 3.56 | 4.47E-03 |
| Nucleoside phosphate binding (GO:1901265) | 2724 | 6 | 1.68 | + | 3.56 | 4.47E-03 |
| Anion binding (GO:0043168) | 2782 | 6 | 1.72 | + | 3.49 | 4.97E-03 |
| Small molecule binding (GO:0036094) | 3138 | 6 | 1.94 | + | 3.09 | 8.96E-03 |
| ATP binding (GO:0005524) | 1954 | 4 | 1.21 | + | 3.31 | 2.88E-02 |
| Adenyl ribonucleotide binding (GO:0032559) | 2033 | 4 | 1.26 | + | 3.18 | 3.27E-02 |
| Adenyl nucleotide binding (GO:0030554) | 2040 | 4 | 1.26 | + | 3.17 | 3.31E-02 |
| Drug binding (GO:0008144) | 2331 | 4 | 1.44 | + | 2.78 | 5.03E-02 |
| Transferase activity (GO:0016740) | 3951 | 4 | 2.44 | + | 1.64 | 2.92E-01 |
| Protein binding (GO:0005515) | 4557 | 4 | 2.82 | + | 1.42 | 5.08E-01 |
| Lyase activity (GO:0016829) | 413 | 3 | 0.26 | + | 11.75 | 1.99E-03 |
| Metal ion binding (GO:0046872) | 3110 | 3 | 1.92 | + | 1.56 | 4.31E-01 |
| Cation binding (GO:0043169) | 3133 | 3 | 1.94 | + | 1.55 | 4.33E-01 |
| Carbon-carbon lyase activity (GO:0016830) | 119 | 2 | 0.07 | + | 27.19 | 2.50E-03 |
| GTP binding (GO:0005525) | 257 | 2 | 0.16 | + | 12.59 | 1.09E-02 |
| Purine ribonucleoside binding (GO:0032550) | 259 | 2 | 0.16 | + | 12.49 | 1.11E-02 |
| Purine nucleoside binding (GO:0001883) | 259 | 2 | 0.16 | + | 12.49 | 1.11E-02 |
| Ribonucleoside binding (GO:0032549) | 263 | 2 | 0.16 | + | 12.3 | 1.14E-02 |
| Nucleoside binding (GO:0001882) | 264 | 2 | 0.16 | + | 12.26 | 1.15E-02 |
| Guanyl ribonucleotide binding (GO:0032561) | 278 | 2 | 0.17 | + | 11.64 | 1.27E-02 |
| Guanyl nucleotide binding (GO:0019001) | 278 | 2 | 0.17 | + | 11.64 | 1.27E-02 |
| Structural molecule activity (GO:0005198) | 534 | 2 | 0.33 | + | 6.06 | 4.25E-02 |
| Protein serine/threonine kinase activity (GO:0004674) | 937 | 2 | 0.58 | + | 3.45 | 1.13E-01 |
| Protein kinase activity (GO:0004672) | 1095 | 2 | 0.68 | + | 2.95 | 1.46E-01 |
| Phosphotransferase activity, alcohol group as acceptor (GO:0016773) | 1248 | 2 | 0.77 | + | 2.59 | 1.79E-01 |
| Kinase activity (GO:0016301) | 1420 | 2 | 0.88 | + | 2.28 | 2.18E-01 |
| Transferase activity, transferring phosphorus-containing groups (GO:0016772) | 1643 | 2 | 1.02 | + | 1.97 | 2.70E-01 |
| Catalytic activity, acting on a protein (GO:0140096) | 2666 | 2 | 1.65 | + | 1.21 | 6.78E-01 |
| Nucleic acid binding (GO:0003676) | 3609 | 2 | 2.23 | - | 0.9 | 1.00E+00 |
| Unclassified (UNCLASSIFIED) | 5083 | 2 | 3.14 | - | 0.64 | 7.54E-01 |
| Beta-N-acetylglucosaminylglycopeptide beta-1,4-galactosyltransferase activity (GO:0003831) | 1 | 1 | 0 | + | > 100 | 1.24E-03 |
| Methionine adenosyltransferase activity (GO:0004478) | 4 | 1 | 0 | + | > 100 | 3.09E-03 |
| Adenosylmethionine decarboxylase activity (GO:0004014) | 5 | 1 | 0 | + | > 100 | 3.70E-03 |
| DNA photolyase activity (GO:0003913) | 5 | 1 | 0 | + | > 100 | 3.70E-03 |
| UDP-galactosyltransferase activity (GO:0035250) | 11 | 1 | 0.01 | + | > 100 | 7.39E-03 |
| Photoreceptor activity (GO:0009881) | 15 | 1 | 0.01 | + | > 100 | 9.84E-03 |
| Pectate lyase activity (GO:0030570) | 26 | 1 | 0.02 | + | 62.22 | 1.66E-02 |
| Carbon-oxygen lyase activity, acting on polysaccharides (GO:0016837) | 26 | 1 | 0.02 | + | 62.22 | 1.66E-02 |
| Galactosyltransferase activity (GO:0008378) | 39 | 1 | 0.02 | + | 41.48 | 2.44E-02 |
| Structural constituent of cytoskeleton (GO:0005200) | 50 | 1 | 0.03 | + | 32.36 | 3.11E-02 |
| Carboxy-lyase activity (GO:0016831) | 71 | 1 | 0.04 | + | 22.79 | 4.36E-02 |
| Carbon-oxygen lyase activity (GO:0016835) | 148 | 1 | 0.09 | + | 10.93 | 8.82E-02 |
| Kinase binding (GO:0019900) | 150 | 1 | 0.09 | + | 10.79 | 8.93E-02 |
| Transferase activity, transferring alkyl or aryl (other than methyl) groups (GO:0016765) | 151 | 1 | 0.09 | + | 10.71 | 8.99E-02 |
| Catalytic activity, acting on DNA (GO:0140097) | 197 | 1 | 0.12 | + | 8.21 | 1.16E-01 |
| Calcium ion binding (GO:0005509) | 257 | 1 | 0.16 | + | 6.29 | 1.48E-01 |
| Signaling receptor activity (GO:0038023) | 284 | 1 | 0.18 | + | 5.7 | 1.62E-01 |
| UDP-glycosyltransferase activity (GO:0008194) | 300 | 1 | 0.19 | + | 5.39 | 1.71E-01 |
| Identical protein binding (GO:0042802) | 302 | 1 | 0.19 | + | 5.36 | 1.72E-01 |
| Structural constituent of ribosome (GO:0003735) | 360 | 1 | 0.22 | + | 4.49 | 2.01E-01 |
| Molecular transducer activity (GO:0060089) | 385 | 1 | 0.24 | + | 4.2 | 2.14E-01 |
| mRNA binding (GO:0003729) | 414 | 1 | 0.26 | + | 3.91 | 2.28E-01 |
| Transferase activity, transferring hexosyl groups (GO:0016758) | 436 | 1 | 0.27 | + | 3.71 | 2.38E-01 |
| Enzyme binding (GO:0019899) | 471 | 1 | 0.29 | + | 3.43 | 2.55E-01 |
| Transferase activity, transferring glycosyl groups (GO:0016757) | 640 | 1 | 0.4 | + | 2.53 | 3.30E-01 |
| ATPase activity (GO:0016887) | 652 | 1 | 0.4 | + | 2.48 | 3.35E-01 |
| Sequence-specific DNA binding (GO:0043565) | 857 | 1 | 0.53 | + | 1.89 | 4.16E-01 |
| Nucleoside-triphosphatase activity (GO:0017111) | 896 | 1 | 0.55 | + | 1.81 | 4.31E-01 |
| Pyrophosphatase activity (GO:0016462) | 946 | 1 | 0.58 | + | 1.71 | 4.49E-01 |
| Hydrolase activity, acting on acid anhydrides, in phosphorus-containing anhydrides (GO:0016818) | 953 | 1 | 0.59 | + | 1.7 | 4.51E-01 |
| Hydrolase activity, acting on acid anhydrides (GO:0016817) | 959 | 1 | 0.59 | + | 1.69 | 4.53E-01 |
| RNA binding (GO:0003723) | 1215 | 1 | 0.75 | + | 1.33 | 5.36E-01 |
| Transporter activity (GO:0005215) | 1415 | 1 | 0.87 | + | 1.14 | 5.93E-01 |
| Oxidoreductase activity (GO:0016491) | 1577 | 1 | 0.97 | + | 1.03 | 1.00E+00 |
| DNA-binding transcription factor activity (GO:0003700) | 1721 | 1 | 1.06 | - | 0.94 | 1.00E+00 |
| Transcription regulator activity (GO:0140110) | 1836 | 1 | 1.13 | - | 0.88 | 1.00E+00 |
| DNA binding (GO:0003677) | 2280 | 1 | 1.41 | - | 0.71 | 1.00E+00 |
| Hydrolase activity (GO:0016787) | 3323 | 1 | 2.05 | - | 0.49 | 7.12E-01 |
